# DNA‐mimic for Specific Surface Functionalization of Zr‐MOFs for Bacterial Targeting

**DOI:** 10.1002/anie.202525762

**Published:** 2026-05-23

**Authors:** Anna Scheeder, Jon Ostolaza‐Paraiso, Andrew G. Baker, Juan F. Blandez, Georgina E. Lindop, Simon M. Fairclough, Ljiljana Fruk, Ioanna Mela, David Fairen‐Jimenez, Clemens F. Kaminski

**Affiliations:** ^1^ Department of Chemical Engineering & Biotechnology University of Cambridge Cambridge UK; ^2^ Department of Material Science and Metallurgy University of Cambridge Cambridge UK; ^3^ Department of Pharmacology University of Cambridge Cambridge UK

**Keywords:** bacteria, metal–organic framework, peptide nucleic acid, post‐synthesis modification

## Abstract

Nanosized metal–organic frameworks (MOFs) are versatile platforms used in biomedical applications due to their high loading capacity, large surface area, and tunable functionality. Without surface modifications, these nanoparticles lack cell specificity and are prone to aggregation and degradation in biological environments, reducing their effectiveness. Surface attachment of DNA via phosphate group coordination to zirconium‐based MOFs improves stability, but DNA binding remains non‐site‐specific due to the abundance of phosphate groups in its backbone, limiting DNA's addressability for further functionalization. To address this issue, we present a novel, significantly faster single‐step approach for the post‐synthesis modification of the external surface of PCN‐222 nanoparticles using an uncharged synthetic mimic of DNA, peptide nucleic acids (PNA). By using phosphate‐modified PNA, we achieve surface functionalization through coordination with the Zr_6_ clusters on the MOF surface. The modification produced monodispersed nanoparticles and resulted in slowed drug‐release kinetics compared to unmodified nanoparticles. PNAs enhanced attachment efficiency and hybridization specificity compared to DNA coatings, allowing subsequent conjugation of protein targeting moieties and enabling bacterial targeting of drug‐loaded MOFs. This work introduces phosphotyrosine‐modified PNA as a superior, single‐step surface coating for PCN‐222, allowing controlled post‐functionalization with single‐stranded DNA (ssDNA) and expanding applications in biomedical and materials science.

## Graphical abstract

We introduce phosphotyrosine‐modified PNA as a superior, single‐step post‐synthesis surface coating for PCN‐222. The DNA mimic allows for controlled post‐functionalization with single‐stranded DNA for the attachment of protein moieties for bacterial targeting.

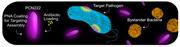

## 1 Introduction

Metal–organic frameworks (MOFs) are highly tunable crystalline porous materials with potential applications ranging from energy to healthcare [1, 2, 3]. These materials are engineered through reticular chemistry by selecting appropriate metal clusters, organic linkers, and synthesis routes, allowing for specific geometries with optimized pore and surface properties [4]. The concept of using MOFs as drug delivery platforms was first introduced by Horcajada et al. in 2006, demonstrating the encapsulation of ibuprofen in MIL‐100(Cr) and MIL‐101(Cr) [5]. Since then, the application of MOFs in nanomedicine has rapidly expanded to include not only drug delivery but also for radiotherapy [6], photodynamic therapy [7], and imaging [8]. The potential of MOFs has stimulated their transition to in vivo experiments in rodents and C. Elegans models [9, 10]. For example, Lin and co‐workers demonstrated the efficacy of combining low doses of x‐ray irradiation with the injection of Hf‐based nanoMOFs to eradicate local tumors in mouse models of breast and colorectal cancer [11]. Moreover, Zeng et al. showcased that the formation of a hyaluronic acid‐based hydrogel with doxorubicin‐loaded ZIF‐90 MOF enhanced tumor growth inhibition in colorectal cancer in vivo [12]. Given the increasing emphasis on clinical translation, rigorous assessment of in vivo biosafety—including immune activation and systemic toxicity—is critical and remains a key requirement in the field. In our case, we have recently demonstrated the biocompatibility of PCN‐222 both ex vivo in human peripheral blood mononuclear cells (PBMCs) and in vivo in animal models (wild mice) [10]. In addition, we have seen how intra‐peritoneal administration of PCN‐222 improves the pharmacokinetics of paclitaxel, the standard of care for pancreatic cancer, reducing metastatic spread and tumor growth in vivo [13]. The rapid progression of MOFs in biomedicine applications is indeed exemplified by the entry of the first MOF, RiMO‐301, using combined radio‐immunotherapy, into human clinical trials [14].

To translate MOFs into healthcare solutions, they must be biocompatible and exhibit optimal hydrochemical and colloidal stability in biological fluids. Additionally, their size and shape need to be carefully controlled, typically below 200 nm, to ensure effective circulation and interaction with cells [15]. The external surface chemistry of these nanoparticles is particularly important for determining their final fate and how they are endocytosed [16]. However, due to phosphate–metal interactions and the creation of protein coronae around them, MOF nanoparticles often suffer from poor colloidal stability in biological buffers and lack specific interactions with cellular targets, limiting their application [17]. To overcome these limitations, post‐synthesis modification (PSM), which was pioneered by Kiang et al. in the covalent transformation of tagged framework linkers, emerges as a possible alternative [18]. In their work, the authors reacted an acid anhydride guest with a host bearing alcohol functionalities, forming an ester while preserving the porous framework structure. However, the term PSM was not utilized until 2007, when Wang and Cohen achieved the first deliberate post synthetic covalent modification by reacting the pendant amine groups of IRMOF‐3 with acetic anhydride [19]. Since its emergence, PSM has been widely used for grafting diverse molecules, including polymers [20, 21], lipids [22, 23, 24], antibodies [25, 26], and oligonucleotides [27, 28, 29, 30], onto the external surface of MOFs [31]. Chen et al. 2024 provides a detailed review of the surface modifications explored for biomedical applications [31]. PSM can not only improve the biocompatibility, stability, and targeted delivery of MOFs, but also preserve their structural integrity and crystallinity [24, 32]. However, many PSM methods are labor‐intensive, involve multiple reactions, require toxic solvents and they can restrict the addition of further functionalities.

**FIGURE 1:**
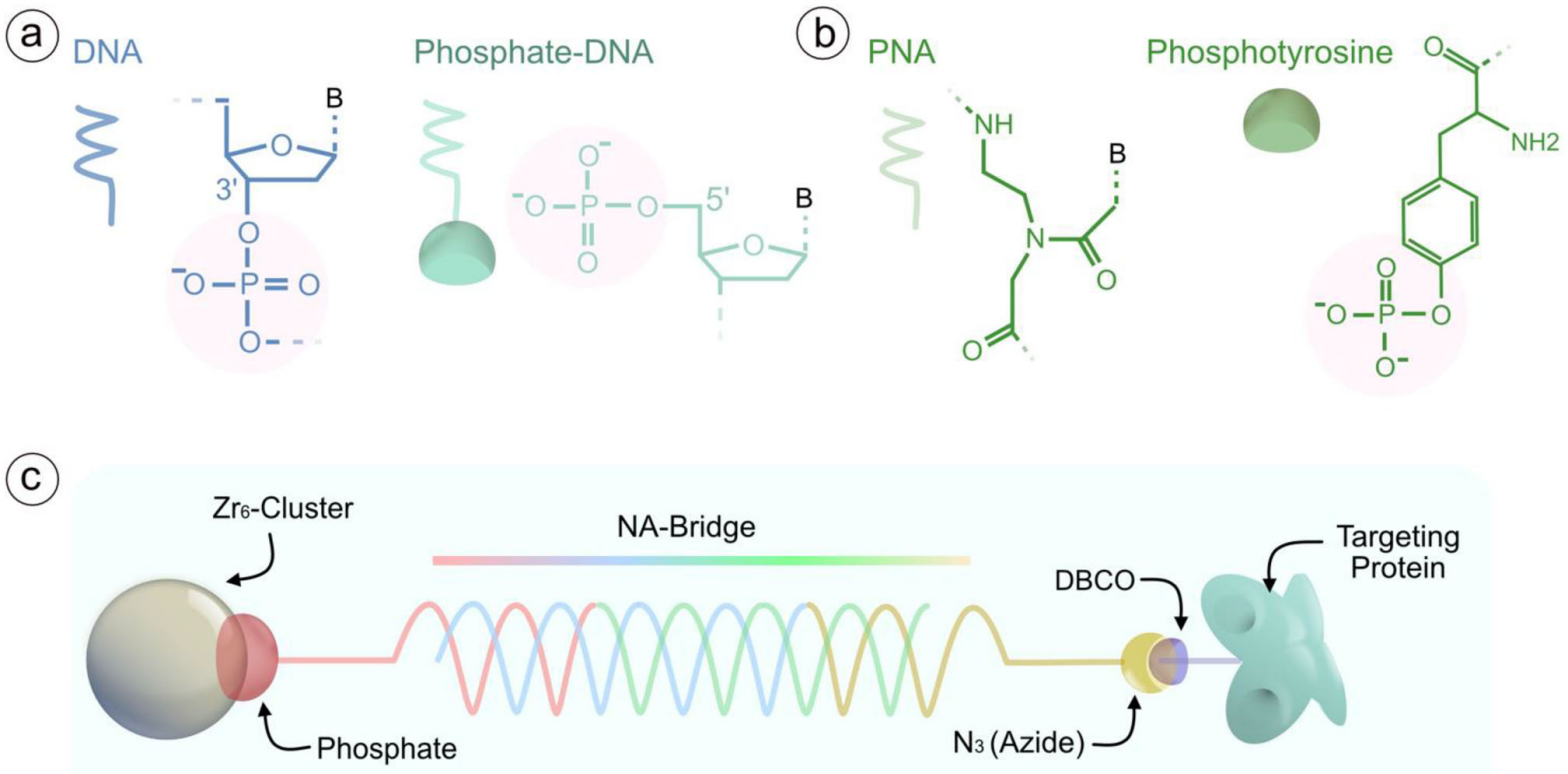
Schematic representation of the post‐synthesis modification of PCN‐222 nanoparticles with nucleic acids for the controlled attachment of bacterial‐specific targeting proteins. Schematic illustration of nucleic acid (NA) backbones of DNA (a) and PNA (b) DNA has a phosphodiester backbone and can carry a terminal phosphomonoester modification while a terminal phosphomonoester modification of PNA is achieved through incorporation of a terminal phosphotyrosine amino acid. (c) Assembly of the targeting complex on PCN‐222 through the coordination of a terminal phosphate monoester (red sphere) of a single stranded nucleic acid (red strand) to an unsaturated Zr_6_ cluster site (gray) on the PCN‐222 nanoparticle's external surface. The nucleic acid is addressable via hybridization with a DNA bridge (blue, green, yellow) carrying a targeting protein (cyan) covalently linked to the DNA through an azide (N_3_) dibenzocyclooctyne (DBCO) reaction.

Several attempts have been reported to decorate the external surface of MOFs [31]. The first macromolecule to be grafted was polyethylene glycol (PEG) [20]. In Horcajada et al. (2009) single step modification process, the amino groups at the terminal end of PEG coordinated with the Fe atoms on the external surface of MIL‐53, MIL‐88, and MIL‐100, coating the external surface of the MOFs with a PEG brush, sterically protecting the nanoparticles from aggregation. This new grafting method through coordination paved the way for further functionalization of the external surface of the MOFs to not only enhance colloidal stability but also add further targeting moieties or imaging agents. In 2015, Wang et al. expanded the functionalization of MOFs to lipids and phospholipids by exploiting the coordination of the terminal phosphate group of 1,2‐dioleoyl‐sn‐glycero‐3‐phosphate (DOPA) to zirconium metal clusters on the external surface of various Zr‐MOFs [22]. Moreover, these lipid molecules can be assembled into bilayers, thereby mimicking mammalian cell membranes, which enhance both the stability and cellular uptake levels of MOFs [23, 24]. In 2021, Chen et al. developed a direct conjugation method to graft PEG onto the external surface of Zr‐MOFs by modifying the terminal end of the PEG chain with a phosphate group [21]. Phosphate‐modified PEG coordinated with the unsaturated Zr atoms forming strong Zr─O─P coordination bonds, effectively anchoring the PEG to the external surface of the MOFs, overcoming the instability of MOFs in phosphate‐rich buffers. Building on this strategy, Siouve et al. developed a phosphate‐PEG conjugation approach to anchor engineered antibody fragments onto the external surface of Zr‐MOFs. This method enabled site‐oriented attachment of Fab fragments for HER2 targeting in breast cancer models, demonstrating that phosphate‐mediated PEG linkers can support selective and stable bioconjugation of targeting ligands while preserving MOF structural integrity [33]. Another widely used and biocompatible PSM approach for MOFs involves the use of DNA [27, 28]. By attaching single‐stranded DNA (ssDNA), the assembly of hybrid nanoclusters was achieved between complementary nanoparticle decorations [29]. ssDNA can be easily grafted onto MOFs, such as zirconium‐based ones like UiO‐66 and PCN‐222, through coordination chemistry between the metal cluster and the phosphate groups of the DNA backbone (Figure 1a) [30]. DNA can bind to MOFs in two distinct modes: “side‐on” via the phosphodiester backbone, or “end‐on”, via the terminal phosphate modification. “Side‐on” binding limits the accessibility of the ssDNA for hybridization with complementary strands, whereas “end‐on” binding leaves the ssDNA accessible for further interaction. Notably, terminal phosphomonoesters exhibit a stronger affinity for coordination with unsaturated metal sites on the external surface of the MOF compared to the phosphates in the phosphodiester backbone, resulting in a PSM that is both accessible and addressable for further hybridization [29]. Yet, while the monoester is preferred, significant competition arises from “side‐on” binding of the diester groups in the backbone alongside with nonspecific van der Waals and electrostatic interactions that also contribute to DNA attachment [29, 34]. The nonspecific and “side‐on” attachment reduces subsequent hybridization of complementary DNA strands, limiting the performance of the overall PSM approach. Wang et al. addressed this issue through PEGylation, which passivates the nanoparticles’ external surface but also enables controlled DNA attachment through chemical linking [34]. To do so, they synthesized several PEG chains with a terminal phosphate group on one end and an azide group on the other. Then, they attached the synthesized PEG chains onto the external surface of the MOF via phosphate–zirconium interactions in DMF, followed by solvent exchange with water. The PEG‐functionalized NPs were finally modified with a DBCO‐TEG modified DNA. However, this approach significantly complicates the design of the PSM procedure, which spans over 5 days with multiple washing cycles between each step, and introduces the use of toxic chemicals such as DMF.

Given the main challenge in DNA grafting stems from the presence of phosphate groups, an important question arises: Could an analogue without a phosphodiester backbone be used for specific ‘end‐on’ attachment? Several xeno‐nucleic acids (XNAs) have been synthetically designed to overcome the limitations of DNA in the past [35, 36]. Among those, peptide nucleic acid (PNA), introduced by Nielsen et al. in 1991 [37], showcases a neutral *N*‐(2‐aminoethyl)‐glycine backbone (Figure 1b), which eliminates the electrostatic repulsion commonly associated with DNA's phosphate backbone. Unlike DNA, PNA's neutrality facilitates more stable interactions with charged surfaces while avoiding the coordination of strong Zr─O─P bonds observed between DNA and Zr‐MOFs. The absence of phosphate groups also allows PNAs to form more stable duplexes with complementary ssDNA sequences without the need for ions to shield charges [38, 39]. Furthermore, PNA, like ssDNA, retains the ability to bind to complementary DNA or RNA sequences through Watson–Crick–Franklin pairing, and its uncharged and flexible backbone often results in higher affinity and specificity compared to natural DNA [39, 40]. Another significant advantage of PNA is its resistance to degradation by nucleases and proteases, which extends the lifetime of the system in biologically relevant environments and offers significant advantages for drug delivery applications [38]. Additionally, the versatility of PNA allows the assembly of chimeric constructs with peptides, linking peptides to plasmids to facilitate the delivery of genetic material [41]. Despite its promise, research on the use of PNA as an external surface modification on MOFs has been limited. Previously, Mejia‐Ariza et al. developed a method to attach PNA to MIL‐88A through a complex synthesis sequence [42]. The process involved the functionalization of the MOF with a biotinylated capping ligand, followed by the non‐covalent attachment of a streptavidin, which then served as a bridge to a previously synthesized biotinylated PNA sequence. While this PNA modification on the MOF's external surface achieved single base mismatch sensitivity in flow cytometry, the procedure was labor‐intensive, limiting its broader adoption.

In this study, we have developed a fast synthesis method for directly grafting PNA onto the external surface of PCN‐222 nanoparticles within 30 min without the need of toxic chemicals such as DMF, exploiting PNA's high affinity and sequence‐specific binding to complementary nucleic acids (Figure 1c). By employing phosphotyrosine‐terminated PNA, we achieved precise surface functionalization via coordination between its terminal phosphate group and Zr_6_ clusters on PCN‐222. For comparison, we also functionalized PCN‐222 with DNA, both with and without terminal phosphomonoester modifications, resulting from either coordination with the phosphates in the DNA backbone or nonspecific electrostatic interactions. Importantly, all modifications produced monodispersed particles with slower drug‐release kinetics compared to unmodified nanoparticles, highlighting their potential to enhance control over delivery. We further assessed attachment efficiency and hybridization specificity of PNA and DNA modifications (both with and without terminal phosphomonoester modifications) with secondary DNA constructs (Figure 1c). We have demonstrated the successful attachment of a targeting protein to drug‐loaded MOFs using a DNA bridge, enabling selective recognition of specific bacteria targets while avoiding nonspecific interactions with bystander bacteria.

## 2 Results and Discussion

### 2.1 Synthesis and Characterization of PCN‐222 and its NA‐Coated Variations

**FIGURE 2:**
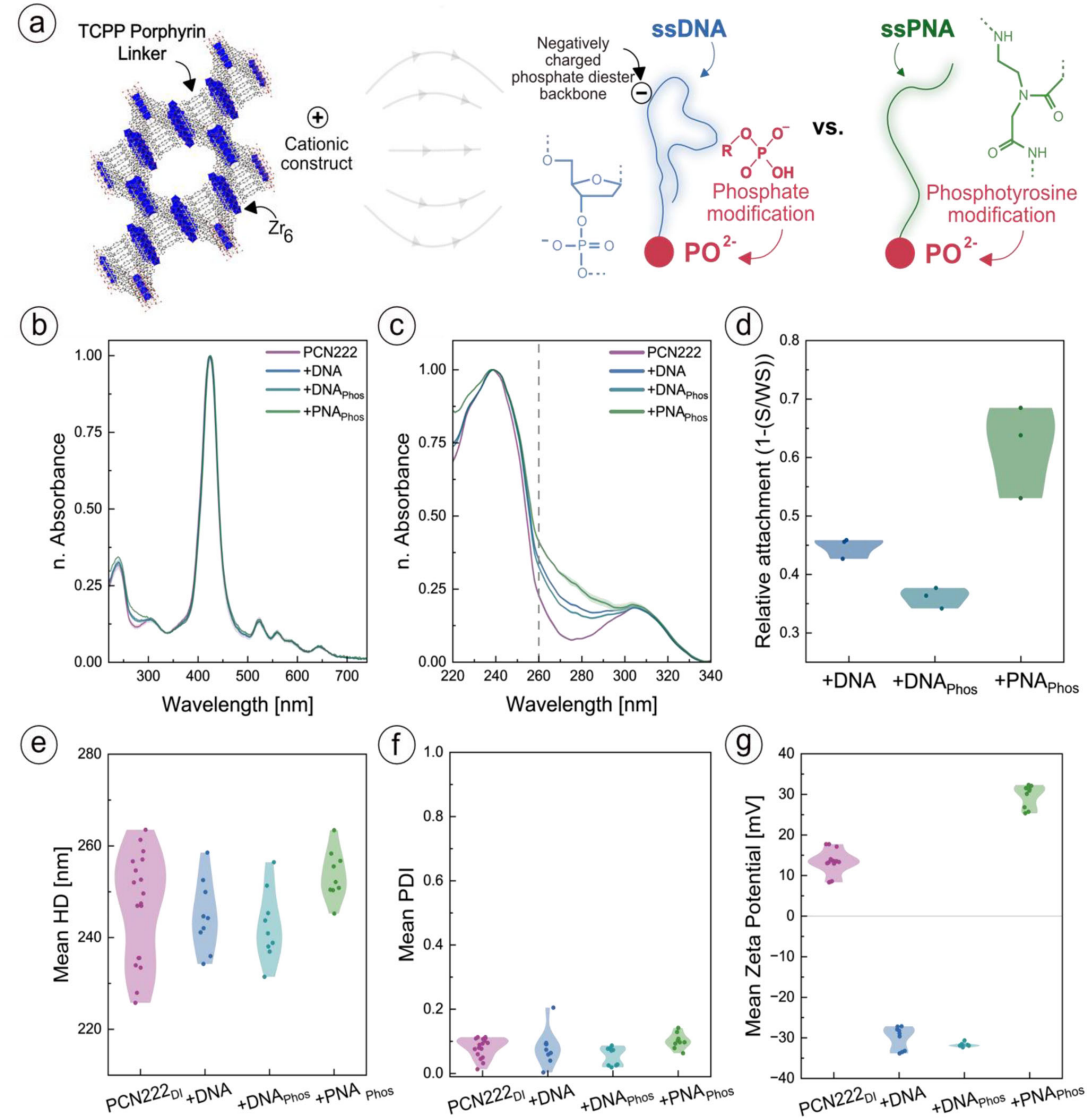
Nucleic acids attach efficiently to the external surface of PCN‐222. (a) Schematic representation of the structure of PCN 222, consisting of cationic Zr_6_ clusters (blue) connected by TCPP linkers (gray). The negatively charged phosphodiester backbone of DNA (blue) facilitates electrostatic interactions with the positively charged Zr_6_ clusters of PCN‐222, while an additional phosphomonoester modification at its terminal provides a specific coordination site. In contrast, PNA (green) with its neutral *N*‐(2‐aminoethyl)‐glycine backbone, minimizes charge‐driven interactions and relies solely on the terminal phosphotyrosine modification for attachment. (b–d). NanoDrop absorption measurements for bare PCN‐222 and NA‐coated material: DNA@PCN‐222 (+DNA), DNAPhos@PCN‐222 (+DNAPhos), and PNAPhos@PCN‐222 (+PNAPhos). (b) Corresponding absorbance spectra. (c) Zoomed‐in detail to show the effect of surface modification on the absorbance spectrum. Dashed line corresponds to the location of the absorbance peak for ssDNA (260 nm). (d) Relative attachment of NAs to PCN‐222. Calculated from the concentration of the NA in the working solution (WS) and supernatant (S). Data collected from three independent measurements. e.g. DLS and zeta potential measurements of PCN‐222 with different NA coatings. The attachment of NA was studied in three independent experiments with each sample being measured by DLS three times. (e) Mean hydrodynamic diameter (HD), and (f) polydispersity index (PDI). (g) Mean zeta potential values.

We synthesized PCN‐222 nanoparticles [21, 43, 44], composed of Zr_6_ clusters connected by tetrakis(4‐carboxyphenyl) porphyrin (TCPP) linkers (Figure 2a), via solvothermal reaction and using trifluoroacetic acid (TFA) as a modulator. Figure S1a shows the powder x‐ray diffraction (PXRD) of the obtained material, with broad peaks at 2.4°, 4.8°, and 7.1°, matching the predicted pattern of the single‐crystal structure and confirming that PCN‐222 was synthesized in high crystallinity. Figure S1b shows the Type I + IV nitrogen adsorption isotherms at 77 K. The first N_2_ molecules fill the triangular micropores (size, 8 nm) together with the formation of the first adsorbed layers on the hexagonal mesoporous channels (size, 3.6 nm) [43, 44]. This resulted in a BET area of 2,031 m^2^/g obtained with BETSI (Figures S2 and S3) [45], consistent with values previously reported in the literature [43]. The resulting nanoparticles were analyzed by scanning electron microscopy (SEM), showing rod‐shaped morphology and an average length of 423 ± 72 nm (mean ± sd, *n* = 100) (Figure S1c). High‐angle‐annular dark‐field scanning transmission electron microscopy (HAADF‐STEM) revealed the presence of lattice fringes, confirming the good crystallinity of the NPs and the presence of mesopores within their structure (Figure S1d).

Dynamic light scattering (DLS) measurements of the bare nanoparticles indicate a smaller average hydrodynamic diameter of 233 ± 3 nm with a polydispersity index (PDI) of <0.1 (Figure S1e). The measured particle sizes can be misleading for non‐spherical particles like PCN‐222, as DLS is sensitive to particle diffusion in solution and uses the inferred diffusion coefficients to determine the hydrodynamic diameter of a similarly behaving spherical particle [46]. Thus, since the technique does not provide an exact size value, it should only be used to study relative size differences between similarly shaped NP systems. Zeta potential measurements of the nanoparticles, following solvent exchange into DI water, showed an initial surface charge of 31.1 ± 0.8 mV (mean ± sd) (Figure S1f), which can be attributed to the positively charged Zr atoms as predominant external surface groups, which is indeed required for nucleic acid coordination. However, the zeta potential drops to 13 ± 3 mV (mean ± sd) and the average hydrodynamic diameter and PDI increased to 256 and 0.1 nm, respectively, when left overnight at room temperature, indicating that bare PCN 222 lacks long‐term colloidal stability in water, consistent with previous observations by Chen et al. (2021) [21].

After confirming the positive external surface potential, we leveraged the availability of unsaturated Zr atoms on the nanoparticle surface to attach phosphate‐bearing nucleic acids, exploiting the strong affinity between zirconium and phosphate groups. We used three types of nucleic acids (NAs): DNA, which contains a phosphate‐rich backbone but lacks terminal phosphate modification; DNA modified with a terminal 3′ phosphomonoester group (DNAPhos); and PNA, characterized by an *N*‐(2‐aminoethyl)‐glycine backbone without internal phosphates but carrying a terminal phosphotyrosine residue for coordination to PCN‐222 (PNAPhos) (Figures 1a and 2a). Supplementary Tables S1.1 and S1.2 show the sequences and their specific modifications.

We functionalized the external surface of PCN‐222 nanoparticles by incubating the NP solution with an excess of either DNA or PNA in nuclease‐free water overnight at room temperature. This resulted in three materials, DNA@PCN‐222, DNAPhos@PCN‐222, and PNAPhos@PCN‐222. After 16 h, we washed the materials with nuclease‐free water to remove unbound NA and collected the supernatant for quantification using a NanoDrop spectrophotometer. PNA and DNA absorb UV light at 260 nm due to presence of purine and pyrimidine rings as part of their bases [47], whereas the absorbance spectrum of PCN‐222 NPs is primarily dictated by their porphyrinic linker TCPP (Figure 2b), but it is also affected by their size [48]. PCN‐222 NPs exhibit fluorescence upon excitation at 647 nm, which we used later to study their localization in fluorescence microscopy. Absorbance spectra of all studied nanoparticles‐PCN‐222 and its PSM forms DNA@PCN‐222, DNAPhos@PCN‐222, and PNAPhos@PCN‐222‐were nearly identical, except that NPs coated with NAs exhibited an additional shoulder in the 260–300 nm range (Figure 2b,c). We attribute this to the presence of PNA or DNA in the system, indicating successful attachment of the NAs to PCN‐222. The absorbance spectrum of PNAPhos@PCN‐222 showed a more prominent shoulder compared to that of PCN‐222, DNA@PCN‐222, and DNAPhos@PCN‐222, suggesting an increase of absorbance observed around 260 nm and, therefore, a higher binding efficiency.

The successful attachment of the NAs to the external surface of PCN‐222 was quantified using the relative attachment value. This value is calculated by the equation 1−[(S)/(WS)] and indicates the fraction of the NA that is no longer in the supernatant (S) compared to its original concentration in the working solution (WS), a larger value implying higher attachment levels of the NAs. A relative attachment value of 0.62 was obtained for PNAPhos@PCN‐222, which is higher than the values of 0.45 and 0.35 obtained for DNA@PCN‐222 and DNAPhos@PCN‐222, respectively. This confirms the successful attachment of all three NAs, and of PNAPhos in particular (Figure 2d). The higher binding efficiency of PNAPhos compared to DNA and DNAPhos is possibly related to its neutral backbone, which avoids attachment to the MOF external surface via electrostatic interactions. Thus, the surface modification of PCN‐222 is likely driven through the coordination with the terminal phosphate group of PNA, resulting in a densely packed “end‐on” attachment configuration. In addition, we speculate that the coordination and electrostatic interactions between the phosphate groups of the DNA backbone and the open metal sites on the MOF lead to the undesired “side‐on” binding of DNA and DNAPhos. This configuration takes more space on the external surface of the nanoparticles than it would if it adopted an “end‐on” configuration, thereby reducing the available binding space for other DNA strands. Moreover, we hypothesize that the overall packing density may be reduced as the highly negative charge of the DNA backbone leads to charge repulsion between neighboring DNA strands, which could account for the lower attachment values compared to PNA. For DNAPhos, the “side‐on” and “end‐on” configurations compete with each other, with “end‐on” attachment via a terminal phosphomonoester previously reported to exhibit higher coordination affinity [29]. Although the NA quantification results suggest reduced binding of DNAPhos in comparison to DNA samples, we would assume increased binding with the “end‐on” attachment. Thus, further assays would be required to fully understand the attachment differences between these samples. These finding highlights a key challenge in achieving NA‐coated Zr‐MOFs based solely on the Zr─O─P coordination between open metal sites of the MOF and terminal phosphate group of DNA. To address this, we pursued various strategies, as discussed in Section S5, including salt ageing procedures (Figure S4) and pH titrations (Figures S5 and S6). While these methods aimed to reduce electrostatic interactions and promote selective Zr─O─P bond formation, nonspecific DNA binding persisted, even under high ionic strength or different pH conditions. Thus, alternative methods such as presented by PNA are required to achieve the desired specificity and functionality.

After confirming NA attachment, we assessed the colloidal stability and surface charge of all coated MOFs. Figure 2e–g show the DLS, PDI, and zeta potential measurements, respectively. All three coated NPs exhibited a single Gaussian size distribution curve with a mean hydrodynamic diameter of ca. 230 nm (Figure S7), with low PDI values (<0.2), indicative of good colloidal stability. Bare PCN‐222 exhibited a positive surface charge of 13 ± 3 mV (mean ± sd), which reversed to −30 ± 2.8 (mean ± sd) and −32 ± 0.5 mV (mean ± sd) for DNA@PCN‐222 and DNAPhos@PCN‐222, respectively, due to their negatively charged phosphate backbones (Figure 2g). In contrast, PNAPhos@PCN‐222 stayed positively charged with 29.5 ± 2.8 mV (mean ± sd), due to the neutral PNA backbone preserving the original positive charge of PCN‐222. All these high absolute values for the three materials contribute to high colloidal stability.

**FIGURE 3:**
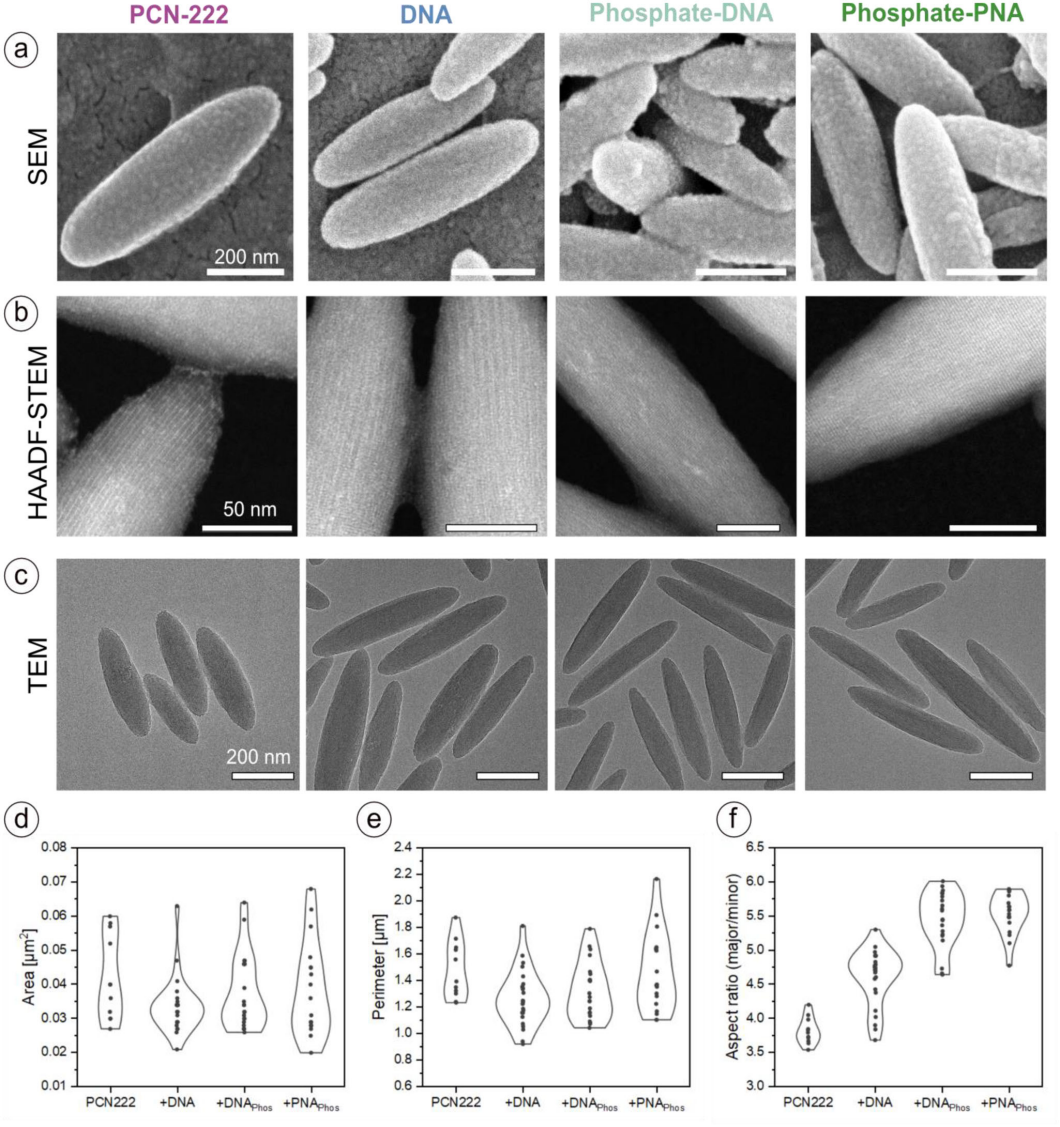
PCN‐222 keeps its crystallinity and rod‐like particle shape after NA coating. (a) Representative SEM, (b) HAADF‐STEM, (c) and TEM images of PCN‐222, DNA@PCN‐222, DNAPhos@PCN‐222, and PNAPhos@PCN‐222 all originating from one initial PCN‐222 synthesis batch. Images in each row are shown to the same scale. (a) SEM images reveal elongated, rod‐shaped morphology of PCN‐222 NPs, which is maintained upon NA coating. (b) HAADF‐STEM images show highly crystalline NPs, evidenced by the presence of lattice fringes. The presence of these lattice fringes in DNA‐, DNAPhos‐, and PNAPhos‐coated PCN‐222 suggests that the crystallinity of the NPs is preserved after the coating process. (c) TEM images show that PSM leads to NP elongation. Extracted particle parameters, that is, area (d), perimeter (e), and aspect ratio (f), are calculated from the ratio between the major and minor axes extracted from a fitted ellipse.

Figure 3 shows the SEM, HAADF‐STEM, and bright‐field transmission electron microscopy (BF‐TEM) images of the nanoparticles. They all show rod‐like particles, with HAADF‐STEM images showing the lattice fringes related to the high crystallinity of the MOF. Importantly, this analysis confirmed that the coating process did not compromise the structural integrity and crystallinity of PCN‐222. Figure 3c–f further show BF‐TEM images of the NPs and their extracted particle dimensions, obtained by fitting the nanoparticles with an ellipse (Figure S9). While the external surface area and perimeter of the NPs remained constant, the length‐to‐width aspect ratio (AR) increased after PSM, observing more elongated shapes upon external surface modification (Figure 3d–f). Since organic NA coatings do not feature sufficient electron density to provide contrast in TEM measurements, the observed shape changes correspond to the nanoparticle itself. Compared to bare PCN‐222, which had an average AR of 3.8 (*n* = 11), the effect was more pronounced in DNAPhos@PCN‐222 (AR = 5.45, *n* = 21) than in DNA@PCN‐222 (AR = 4.6, *n* = 25), potentially caused by the presence of additional terminal phosphate monoester. However, the strongest effect was seen with PNAPhos@PCN‐222 (AR = 5.5, *n* = 18) which only exhibits a single terminal phosphate group. Since the volume of the nanoparticles remained constant during elongation, the results indicate a reshaping process rather than corrosion or material loss. We also observed that the elongation of nanoparticles increased with DNAPhos concentration (Figure S10). These results were unexpected, as PCN‐222 is considered a stable material and has not been previously reported to exhibit post‐synthetic shape change or flexibility. FFT analysis of HAADF‐STEM images revealed consistent lattice spacings characteristic of PCN‐222 across all modified and drug‐loaded samples, confirming preservation of the crystalline framework and, therefore, excluding any form of recrystallization or phase transformation upon coating (Figure S11) [48]. The difference between the three different NAs used further suggests that the terminal phosphomonoester enhances surface rearrangement on the system. However, if the interaction between phosphates and the Zr_6_ nodes were strong enough to break the TCPP‐Zr_6_ bond, particle dissolution would be expected, which we did not observe, strongly supporting a reshaping mechanism rather than recrystallization, corrosion, or degradation. Understanding the driving forces behind this morphological transformation, including potential surface energy differences and interaction forces, would require further investigation. Beyond NP characterization, the cell viability with surface‐coated PCN‐222 nanoparticles was tested on the human‐derived fibroblasts cell line Wi‐38. PNAPhos functionalized PCN‐222 significantly reduced cytotoxicity compared with bare and DNA‐coated nanoparticles (Figure S12), further highlighting that more controlled surface functionalization likely driven by terminal phosphate monoester modification enhances the biocompatibility of PCN‐222.

Figure S8 shows the energy dispersive x‐ray spectroscopy (EDS) images, identifying the elemental composition of the NPs. PNAPhos@PCN‐222 had a higher nitrogen content (7.17%) than DNA@PCN‐222 (3.99%) and DNAPhos@PCN‐222 (3.72%), which we attribute to PNA's *N*‐(2‐aminoethyl)‐glycine backbone. On the other hand, DNA@PCN‐222 and DNAPhos@PCN‐222 displayed higher phosphate levels. The P/Zr ratio further highlighted coating differences between the DNA samples, with DNA@PCN‐222 showing a higher value (0.33) than DNAPhos@PCN‐222 (0.25), suggesting higher attachment of DNA in comparison to DNAPhos, consistent with NanoDrop quantifications (Figure 2c–e).

### 2.2 PNA Surface Modifications Reduce Nonspecific Hybridization of DNA Assemblies

**FIGURE 4:**
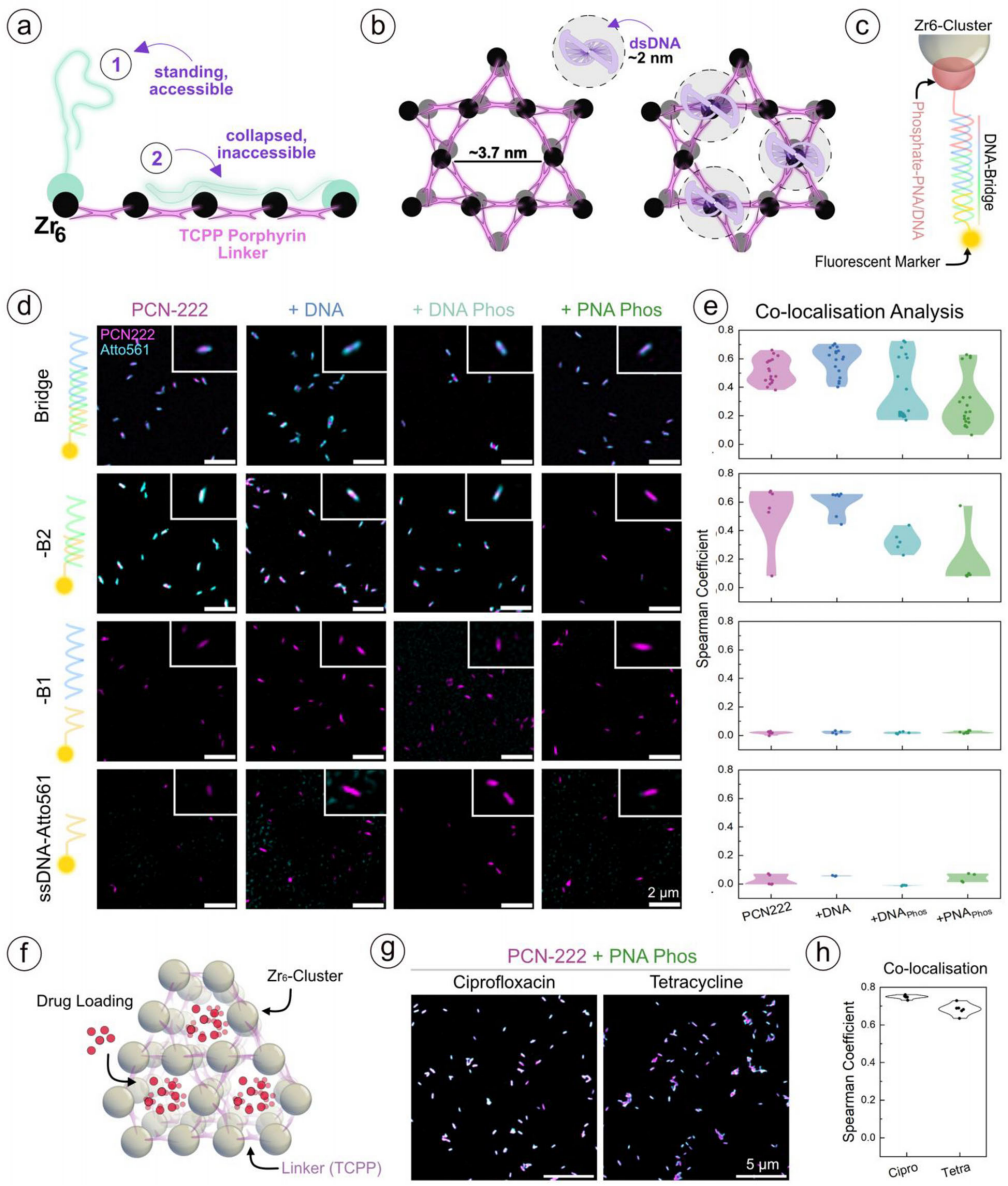
PNAPhos coating retains its specific hybridization capabilities with Atto‐Bridge after encapsulation of antimicrobials. (a) Schematic illustration of NA attachment to external surface of PCN‐222, showing configurations where NA is either freely accessible for hybridization with complementary ssDNA sequences, or collapsed and inaccessible for further modification. (b) Schematic of a PCN‐222 unit cell, comparing the spacing between Zr_6_ clusters and the width of dsDNA (∼2 nm), indicating potential steric hindrance preventing dsDNA from occupying all Zr sites. (c) Schematic depiction of the Atto‐Bridge component linking an Atto561 fluorophore to the MOF external surface. The Atto‐Bridge comprises ssDNA carrying an Atto561 dye (ssDNA‐Atto561), a 40‐mer ssDNA sequence binding to the dye strand (B1), and a second 40‐mer sequence (B2) designed to hybridize with the surface modification on PCN‐222 and B1. (d) Representative structured illumination microscopy (SIM) images showing co‐localization of PCN‐222 (magenta) with the Atto‐Bridge (cyan). Control experiments lacking B1 (‐B1), B2 (‐B2), or using only Atto561‐modified ssDNA were performed. (e) Co‐localization analysis between MOF and the Atto561 dye using the Spearman coefficient method. (f) Schematic of drug‐loaded PCN‐222, indicating loading into the mesoporous network. (g) Representative SIM images showing co‐localization of drug‐loaded and PNAPhos‐coated PCN‐222 (magenta) with the Atto‐Bridge (cyan). (h) Co‐localization analysis between ciprofloxacin‐ (Cipro) and tetracycline‐ (Tetra) loaded MOF and the Atto561 dye using the Spearman coefficient method.

After confirming the successful coating of all NAs on the external surface of the MOFs, we assessed the accessibility of these NAs to hybridize with complementary DNA strands. As described above, we hypothesized the PNA coating would adopt an accessible conformation due to “end‐on” attachment, whereas both DNA coatings were expected to be predominantly inaccessible due to the “side‐on” attachment (Figure 4a). However, even if the attachment is better controlled for PNA, its accessibility for hybridization with complementary ssDNA strands may still be limited due to steric hindrance between the coordinated PNA strands. The Zr clusters in PCN‐222 are spaced by TCPP linkers of approximately 2 nm length [49], restricting hybridization due to insufficient spacing, as a DNA double helix is ∼2 nm wide (Figure 4b) [50]. To study the hybridization capabilities, we tested and compared the attachment efficiency of a DNA construct, which we referred to as the “Bridge”, across the three different surface coatings. The Atto‐Bridge (BA) we designed consists of three ssDNA sequences (Table S1.2): one carrying an Atto561 dye for visualization using fluorescence microscopy (ssDNA Atto561), a 40‐mer ssDNA sequence binding to the dye strand (B1), and a second 40‐mer sequence (B2) designed to hybridize with the surface modification on PCN‐222 and B1 (Figure 4c). The Atto‐Bridge assembly was confirmed via gel electrophoresis (Figure S14). Next, the construct was incubated with bare and coated PCN 222 nanoparticles and the specificity of the attachment was assessed using structured illumination microscopy (SIM), a super‐resolution microscopy technique of ca. 120 nm resolution. The attachment was confirmed through a Spearman co‐localization analysis between the fluorescence signal of PCN‐222's inherent fluorescence (647 nm excitation, magenta) and the Atto561 signal (561 nm excitation, cyan). Figure 4d shows representative SIM images for bare and NA‐coated PCN‐222 after incubation with the Atto‐Bridge, showing co‐localization between the MOF and the bridge for all systems (Figure 4d, top row). Co‐localization was observed even with bare PCN‐222, which lacks any sort of DNA coating, suggesting that electrostatic interactions between the negatively charged DNA‐based Atto‐Bridge and the positively charged PCN‐222 played a major role in the external surface attachment. When the MOFs were incubated with a truncated Atto‐Bridge lacking B2 (“Atto‐Bridge—B2”), specific attachment of the Atto‐Bridge to the MOFs’ external surface should be prevented. However, SIM images revealed significant co‐localization for the bare MOF, DNA‐ and DNAPhos‐coated PCN‐222, with similar Spearman coefficients for the full Atto‐Bridge and the truncated Atto‐Bridge, suggesting non‐specific binding (Figure 4e). DNAPhos‐coated PCN‐222 showed reduced co‐localization compared to the full Atto‐Bridge, but it remained significant (Figure 4e, second row). In contrast, PNAPhos‐coated PCN‐222 exhibited minimal co‐localization with the truncated Atto‐Bridge, confirming that Atto‐Bridge binding occurs primarily through specific hybridization in the PNA‐coated samples. Finally, when incubated with another truncated Atto‐Bridge missing B1 (“Atto‐Bridge—B1”) or just the ssDNA‐Atto561 dye strand, none of the materials showed co‐localization, indicating that electrostatic interactions between ssDNA‐Atto561 and the nanoparticles were insufficient for binding. These results suggest that the PNA coating provides the best external PSM for an accessible and sequence‐specific attachment of complementary sequences. The difference between DNA and DNAPhos modifications, with DNAPhos enabling more specific attachment, aligns with previous findings by Wang et al. (2017) [29]. Their study demonstrated preferred coordination between the cationic metals of the nanoparticles and the terminal phosphomonoester modification of DNA compared to the phosphodiester backbone. This preferential binding results in more “end‐on” modifications of the MOF, theoretically improving the accessibility of complementary DNA constructs.

### 2.3 Drug Encapsulation Does not Affect NA Assembly on the MOF External Surface

Once the accessibility of PNA on the MOF surface was confirmed, we investigated whether drug encapsulation would impact the NA attachment and subsequent assembly of the Atto‐Bridge (Figure 4f). Therefore, we used the model antibiotics ciprofloxacin and tetracycline, both of which exhibit distinct UV–vis absorption peaks (Figure S16). Ciprofloxacin and tetracycline were chosen as the antimicrobial agents due to their well‐studied nature, widespread commercialization, and intracellular‐acting nature, meaning they need to be delivered inside the bacterial membrane to be effective [51, 52]. The amount of drug loaded in PCN‐222 was determined by subtracting the drug concentration in the supernatant from the initial drug concentration in the working solution, using a previously established calibration curve in the corresponding reaction solvent (Figure S17). Ciprofloxacin and tetracycline were successfully loaded into PCN‐222 in water and ethanol, respectively, achieving drug loadings of 41% (*n* = 3) for ciprofloxacin (Ci@PCN‐222) and 51% (*n* = 3) for tetracycline (Te@PCN‐222). These loading values were obtained by dividing the amount of drug loaded by the total weight of the system. Next, we studied the release kinetics of ciprofloxacin. This study aimed to provide an initial comparative assessment of how the PNAPhos coating influences drug release behavior. The experiments were only carried out with ciprofloxacin due to the limited solubility of tetracycline in PBS. The release kinetic studies of ciprofloxacin were carried out from both uncoated (Ci@PCN‑222) and PNAPhos‐coated PCN‐222 (PNAPhos@Ci@PCN‐222) by resuspending the nanoparticles in PBS (Figure S18). Although these experiments were conducted as single measurements, the data suggest a slower release profile for the PNAPhos‐coated PCN‐222. The initial rapid release observed in both systems likely corresponds to ciprofloxacin adsorbed on the external surface of the MOF, while the subsequent slower release may be attributed to the drug being gradually released from the internal pores of the MOF as the MOF structure degrades in the phosphate buffer. The reduced release rate observed from the coated PCN‐222 is likely due to the coating slowing down the degradation of the MOF, thereby delaying drug release. Following the release studies, we tested the effect of drug encapsulation on the accessibility of the PNAPhos coating for its subsequent Atto‐Bridge hybridization. Post coating, both PNAPhos@Ci@PCN‐222 and PNAPhos@Te@PCN‐222 were incubated with the Atto‐Bridge, and their co‐localization was analyzed using SIM. Figure 4g shows the SIM images from PNA‐coated and drug loaded MOFs after incubation with the Atto‐Bridge, with both PNAPhos@Ci@PCN‐222 (magenta, left) and PNAPhos@Te@PCN‐222 (magenta, right) displaying significant co‐localization with the Atto‐Bridge (cyan), as indicated by the overlapping blue and magenta regions. Figure 4h shows the quantification of the co‐localization using the Spearman coefficient. The high values obtained in both PNAPhos@Ci@PCN‐222 and PNAPhos@Te@PCN‐222 confirm that drug loading did not interfere with the PNAPhos attachment on the external surface of the PCN‐222 nor with the subsequent Atto‐Bridge assembly.

### 2.4 PNA Coating Facilitates Assembly of Bacterial Targeting Complex

**FIGURE 5:**
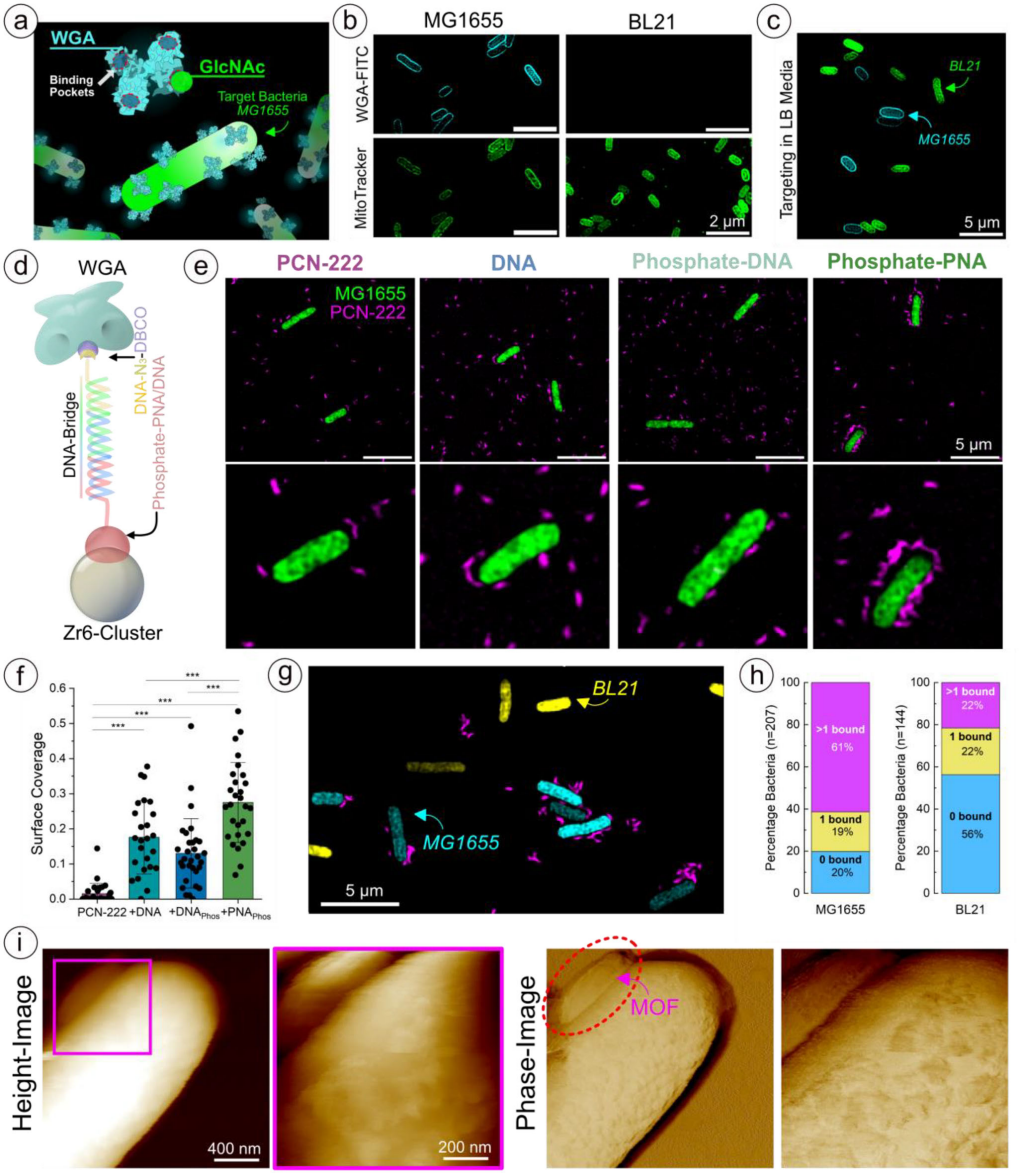
Cell‐specific targeting via assembly of a protein targeting moiety on PNA‐coated PCN‐222. (a) Schematic representation of WGA, the targeting protein, binding to its target, MG1655 bacteria, which feature a terminal *N*‐acetylglucosamine sugar in their LPS layer. (b) Representative SIM images show bacterial targeting by WGA. MG1655 bacteria (green) were labeled with MitoTracker to stain their membranes, followed by targeting with FITC‐labelled WGA (cyan) (c) Representative SIM images showing bacterial targeting by WGA in a co‐culture of MG1655 and BL21. (d) Schematic illustration of the targeting WGA‐Bridge showing the attachment of the sugar binding protein WGA, to the MOFs surface. (e) Representative SIM images of PCN 222 (magenta) and MG1655 bacteria (green) after incubation with bare and NA‐coated PCN‐222. (f)Quantification of fluorescence microscopy data to determine PCN‐222 surface coverage of MG1655 cells. Box plots display data with whiskers extending to 1 standard deviation from the mean; n ≥ 25 cells per condition were analyzed. Statistical analysis was performed with a one‐way ANOVA with Bonferroni's multiple comparison test (*p < 0.05, **p < 0.01, ***p < 0.001). (g) Representative SIM image of a bacterial co‐culture showing BL21 (yellow) and MG1655 (cyan) bacteria incubated with WGA@PNAPhos@T@PCN‐222. The drug‐loaded, coated MOFs specifically targeted MG1655 cells, while BL21 cells remained untargeted. (h) Comparison of targeting efficiency for MG1655 and BL21 cells. Interactions were quantified from fluorescence microscopy images by assessing MOF association with each bacterial strain, categorizing cells into three groups: no detectable MOF fluorescence signal in their vicinity (“0 bound”), association with a single nanoparticle (“1 bound”), and association with more than one MOF nanoparticle (“>1 bound”). In total, 207 MG1655 bacteria and 144 BL21 cells were analyzed. I. Height (left) and phase (right) AFM images of MG1655 bacteria targeted by WGA‐decorated PNAPhos‐coated MOF. The bacterial membrane at the MOF interface remains intact, indicating that the MOF does not visibly affect membrane integrity.

To test whether the nanoparticles could be used for cell targeting, we designed a targeting complex named “WGA‐Bridge” for selective delivery of the modified PCN‐222 to MG1655 E. coli. This complex utilized a DNA Bridge modification to attach wheat germ agglutinin (WGA), a 36 kDa protein specific to *N*‐acetylglucosamine (GlcNAc), the terminal lipopolysaccharide (LPS) sugar in MG1655 (Figure 5a). First, the targeting capabilities of WGA were tested against GlcNAc‐containing bacteria (MG1655) and GlcNAc‐lacking cells (BL21). To do so, FITC‐labeled WGA was incubated with MitoTracker‐stained MG1655 and BL21 separately, and the co‐localization was analyzed by SIM. Figure 5b shows the SIM images of FITC‐labeled WGA (cyan) and MG1655 and BL21 bacteria (green); The images show an overlap of WGA (cyan) and membrane (green) fluorescence for MG1655 but not for BL21, confirming the successful binding of WGA to MG1655 but not to BL21 due to the presence and lack, respectively, of GlcNAc in their LPS layers. Figure S13d shows the co‐localization analysis between the bacterial cells and the WGA using the Spearman coefficient. This analysis confirms the increased co‐localization between MG1655 and WGA. Moreover, when the experiments were carried out in the presence of GlcNAc in solution (+GlcNAc), the co‐localization reduced significantly, further confirming the specificity of the process. The toxicity of the protein to the bacteria was analyzed by monitoring the growth rate of MG1655 cells at different WGA concentrations. Figure S13e shows the evolution of the bacterial growth rate upon addition of different amounts of WGA, and the lack of change in its growth rate confirms the nontoxic nature of the targeting protein. Figure 5c shows the SIM images of a bacteria co‐culture of both MG1655 and BL21 with FITC‐labelled WGA. MG1655 cells were not stained, whereas BL21 cells were stained with MitoTracker Deep Red. The image confirms the selective targeting of MG1655, demonstrating further its capabilities as a precise targeting moiety.

After confirming the targeting capabilities of the protein, we synthesized a WGA‐Bridge that would bind to the NA coating on the external surface of PCN‐222 following the same principle as previously described for the Atto‐Bridge. Figure 5d shows the description of the strategy. The WGA‐bridge consisted of the hybridized product of the previously ssDNA‐conjugated WGA with complementary DNA sequences B1 and B2. As a first step, WGA was conjugated to a ssDNA sequence via DBCO‐azide click chemistry and confirmed by SDS‐PAGE. Figure S14c shows the acrylamide gel of WGA‐ssDNA. In the first lane, which corresponds to only WGA, a single band is observed at 18 kDa, representing one monomer of WGA. The appearance of a second and third band in lane 2 and 3 at 28 and 38 kDa indicates the successful binding of one and two ssDNA sequences, respectively, as the observed increase corresponds to the molecular weight of the nucleic acid. Then, the targeting capabilities and specificity of the targeting complex upon conjugation to ssDNA and further hybridization with complementary DNA sequences were assessed via SIM by conjugating a fluorescent marker at the other end of the bridge. Figure S15 shows the assembly of the targeting complex with WGA after its conjugation with a ssDNA sequence. MG1655 cells were incubated with the WGA‐Bridge and three control experiments: the WGA‐Bridge without B2, the Atto‐Bridge, and the WGA‐ssDNA. The significant overlap observed between MG1655 cells (green) and the targeting complex (magenta), and the subsequent co‐localization analysis confirms that the targeting capabilities of the protein were retained after conjugating a ssDNA sequence (Figure S15b,c). Moreover, the lack of fluorescence observed when B2 was missing or when only the Bridge was added, confirms not only that targeting only occurs in the presence of WGA, but also that all components in the formation of the Bridge were necessary. Here, WGA serves as a proof‐of‐concept targeting moiety, proven to bind GlcNAc‐containing bacteria (MG1655). However, WGA could be replaced with other proteins or aptamers specific to target cells. Aptamers would enable easier attachment to nucleic acid‐modified nanoparticles through direct hybridization, avoiding the need to conjugate proteins to DNA sequences. In addition to aptamer‐based strategies, alternative targeting moieties could be attached via click chemistry by either directly introducing the reactive groups onto the linker, or by functionalizing an intermediate molecule (e.g., PMA) to carry click‐compatible functionalities [53, 54].

We next assembled the WGA‐Bridge on the external surface of the bare and NA‐coated MOFs using the same procedure as previously described for the Atto‐Bridge. After the WGA‐Bridge assembly, we incubated the modified nanomaterials with SYTO 9‐stained live MG1655 bacteria (green) (Figure 5e). Figure 5e shows representative SIM images of bacterial samples exposed to WGA Bridge‐decorated PCN‐222, DNA@PCN‐222, DNAPhos@PCN‐222, and PNAPhos@PCN‐222. Bare PCN‐222 (magenta) exhibited minimal targeting, with only weak attachment to bacteria, suggesting that binding was primarily due to nonspecific interactions (Figure 5f). On the other hand, DNA@PCN‐222 and DNAPhos@PCN‐222 (magenta) attached more efficiently to the bacterial cell surface, indicating some bacterial targeting (Figure 5f). In contrast, PNAPhos‐coated PCN‐222 displayed visibly more attachment to MG1655, as evidenced by the accumulation of MOF NPs around the bacteria, creating the appearance of a halo effect (Figure 5f). The observed gap between the nanoparticles and cytoplasmic staining is attributed to the unstained bacterial cell envelope [Scheeder 2023], indicating successful hybridization of the WGA‐Bridge with the PNAPhos sequence on the MOF, thus achieving effective targeting of MG1655 bacteria. All in all, the presence of the phosphate group in DNAPhos@PCN‐222 did not visibly enhance specificity, further suggesting that binding was not primarily driven by the intended hybridization mechanism. Overall, PNAPhos‐coated PCN‐222 was the most effective in targeting MG1655 via the WGA Bridge, while other coatings led to nonspecific binding and less effective targeting.

We further assessed the targeting capabilities upon antibiotic encapsulation. Here, we assembled again the WGA‐Bridge on tetracycline‐loaded and PNAPhos‐coated nanoparticles (WGA@PNAPhos@Te@PCN‐222) and incubated them with a co‐culture of BL21 and MG1655 bacteria (Figure 5g). BL21 cells were stained with MitoTracker orange (yellow) and MG1655 with SYTO 9 (cyan) before incubation with WGA@PNAPhos@Te@PCN‐222. SIM images of the bacterial co‐culture (Figure 5g) confirmed that WGA‐conjugated PNAPhos‐coated PCN‐222 predominantly targeted MG1655 even after drug encapsulation, as the MOF was located primarily around the SYTO 9‐stained bacteria (Figure 5h). These results validate the specificity of the targeting mechanism in a mixed bacterial environment, demonstrating the potential for targeted drug delivery in complex settings.

To explore if MOF attachment impacted cell envelope integrity, we used atomic force microscopy (AFM) to visualize the topography of MG1655 cells upon nanoparticle targeting. The cell envelope appeared intact after 30 min of incubation with the MOFs (Figure 5i). This, together with the kinetic profiles of drug release from the MOFs could explain the fact that no significant difference was observed in the bactericidal or bacteriostatic effects between drug‐loaded PCN‐222 with and without the targeting complex (Figure S21). Slow drug release, while perhaps not compatible with the rapid replication rates of planktonic bacteria, could offer a very useful tool against bacteria living in biofilms, in habitats shared with commensal bacteria. Moreover, since tetracycline acts intracellularly, targeting the cell envelope may not guarantee effective drug delivery to the cytoplasm.

## 3 Conclusion

We evaluated the surface modification of PCN‐222 with various nucleic acids to create an accessible platform for further functionalization, such as hybridization with the DNA‐conjugated protein WGA. The negatively charged phosphate backbone of DNA competes with the formation of specific Zr─O─P bonds due to electrostatic interactions with the positively charged unsaturated Zr atoms on PCN‐222, necessitating alternative methods to achieve enhanced specificity. Thus, we tested PNA as an alternative to DNA, as its neutral backbone minimizes non‐specific electrostatic interactions, potentially improving targeting efficiency and specificity in MOF‐based delivery systems.

Our PNAPhos modification demonstrated higher binding efficiency to PCN‐222 than DNA and DNAPhos, while maintaining the crystallinity and colloidal stability of the nanoparticles. These PNAPhos‐coated PCN‐222 nanoparticles showed the highest specificity in co‐localization with a complementary Atto‐Bridge, as negative controls revealed no significant co‐localization. In contrast, DNA‐ and DNAPhos‐coated PCN‐222 displayed co‐localization with both the Atto‐Bridge and some negative controls, indicating that electrostatic interactions contributed to non‐specific binding.

Next, we tested the assembly of a targeting moiety on the PNAPhos‐coated nanocarriers surface and found that the hybridization of the coated MOFs with the WGA‐Bridge resulted in pronounced and specific targeting of MG1655 cells. Moreover, drug loading did not affect PNA attachment or subsequent targeting of MG1655 bacteria, while control BL21 bacteria remained untargeted. With the modular DNA bridge design, individual components such as the targeting moieties can be easily switched out for a different application. Here, however, the enhanced targeting did not translate to improved bactericidal effects when compared to non‐targeted nanoparticles. This observation was attributed to two factors: (1) working with planktonic bacteria in liquid culture results in naturally high collision frequencies between bacteria and nanoparticles at saturating concentrations, (2) the slow release of the drug from the MOF porous matrix, which, while beneficial for sustained release, limits efficacy against planktonic bacteria, and (3) the intracellular‐acting nature of the antimicrobial, which may not benefit significantly from increased targeting. While the drug‐loaded nanoparticles did not enhance antibiotic activity in planktonic bacterial assays, smaller‐sized, targeted MOFs with PNA or DNA coatings could function as nanoantibiotics themselves. Recent work by Jiang et al. demonstrated that nanoparticles with hydrophilic brush coatings exhibit size‐dependent antibiotic activity, effectively disrupting bacterial membranes only when their size is below 50 nm [55]. Furthermore, the slow‐release profile of these nanoparticles may offer advantages in biofilm applications, where bacteria persist within a dense, hard‐to‐penetrate matrix. Future experiments should therefore focus on evaluating nanoparticle efficacy across different sizes, particularly within the context of biofilms.

In conclusion, we present a versatile surface coating that, through its charge neutrality, allows the specific attachment of ssDNA capable of carrying additional functionalities.

## Conflicts of Interest

D.F.J. is cofounder of Vector Bioscience Cambridge, working on the commercialization of MOFs in healthcare applications.

## Supporting information



The authors have cited additional references within the Supporting Information [21, 29, 37, 40, 45, 56, 57, 58, 59, 60, 61, 62, 63].
**Supporting File**: anie72381‐sup‐0001‐SuppMat.docx.

## Data Availability

The data that support the findings of this study are available in the supplementary material of this article.

## References

[1] J. R. Li , R. J. Kuppler , and H. C. Zhou , “Selective Gas Adsorption and Separation in Metal–Organic Frameworks,” Chemical Society Reviews 38 (2009): 1477–1504, 10.1039/B802426J. 19384449

[2] W. Gong , Y. Liu , H. Li , and Y. Cui , “Metal‐Organic Frameworks as Solid Brønsted Acid Catalysts for Advanced Organic Transformations,” Coordination Chemistry Reviews 420 (2020): 213400, 10.1016/j.ccr.2020.213400.

[3] A. Wang , M. Walden , R. Ettlinger , et al., “Biomedical Metal–Organic Framework Materials: Perspectives and Challenges,” Advanced Functional Materials 34 (2024): 2308589, 10.1002/adfm.202308589. PMC7617264 39726715

[4] O. M. Yaghi , “Reticular Chemistry: Molecular Precision in Infinite 2D and 3D,” Frontiers Journal 3 (2019): 66–83, 10.1142/S2529732519400054.

[5] P. Horcajada , C. Serre , M. Vallet‐Regí , M. Sebban , F. Taulelle , and G. Férey , “Metal–Organic Frameworks as Efficient Materials for Drug Delivery,” Angewandte Chemie International Edition 45 (2006): 5974–5978, 10.1002/anie.200601878. 16897793

[6] Z. Xu , W. Zhen , C. McCleary , et al., “Nanoscale Metal–Organic Framework With an X‐Ray Triggerable Prodrug for Synergistic Radiotherapy and Chemotherapy,” Journal of the American Chemical Society 145 (2023): 18698–18704, 10.1021/jacs.3c04602. 37581644 PMC10472429

[7] X. Chen , B. B. Mendes , Y. Zhuang , et al., “A Fluorinated BODIPY‐Based Zirconium Metal–Organic Framework for In Vivo Enhanced Photodynamic Therapy,” Journal of the American Chemical Society 146 (2024): 1644–1656, 10.1021/jacs.3c12416. 38174960 PMC10797627

[8] I. A. Lázaro , X. Chen , M. Ding , et al., “Metal–Organic Frameworks for Biological Applications,” Nature Reviews Methods Primers 4 (2024): 42, 10.1038/s43586-024-00320-8.

[9] S. Rojas , T. Hidalgo , Z. Luo , D. Ávila , A. Laromaine , and P. Horcajada , “Pushing the Limits on the Intestinal Crossing of Metal–Organic Frameworks: An Ex Vivo and In Vivo Detailed Study,” ACS Nano 16 (2022): 5830–5838, 10.1021/acsnano.1c10942. 35298121 PMC9047668

[10] Y. Zhuang , B. B. Mendes , D. Menon , et al., “Multiscale Profiling of Nanoscale Metal‐Organic Framework Biocompatibility and Immune Interactions,” Advanced Healthcare Materials 14 (2025): e01809, 10.1002/adhm.202501809. 40772350 PMC12616609

[11] K. Lu , C. He , N. Guo , et al., “Low‐Dose X‐Ray Radiotherapy–Radiodynamic Therapy via Nanoscale Metal–Organic Frameworks Enhances Checkpoint Blockade Immunotherapy,” Nature Biomedical Engineering 2 (2018): 600–610, 10.1038/s41551-018-0203-4. 31015630

[12] Y. Zeng , C. Zhang , D. Du , et al., “Metal‐Organic Framework‐Based Hydrogel With Structurally Dynamic Properties as a Stimuli‐Responsive Localized Drug Delivery System for Cancer Therapy,” Acta Biomaterialia 145 (2022): 43–51, 10.1016/j.actbio.2022.04.003. 35398545

[13] F. Melle , D. Menon , J. Conniot , et al., “Rational Design of Metal–Organic Frameworks for Pancreatic Cancer Therapy: From Machine Learning Screening to In Vivo Efficacy,” Advanced Materials 37 (2025): e2412757, 10.1002/adma.202412757. 39895194 PMC12747470

[14] M. Koshy , M. Spiotto , L. E. Feldman , et al., “A Phase 1 Dose‐Escalation Study of RiMO‐301 With Palliative Radiation in Advanced Tumors,” Journal of Clinical Oncology 41 (2023): 2527–2527, 10.1200/JCO.2023.41.16_suppl.2527.

[15] J. Dolai , K. Mandal , and N. R. Jana , “Nanoparticle Size Effects in Biomedical Applications,” ACS Applied Nano Materials 4 (2021): 6471–6496, 10.1021/acsanm.1c00987.

[16] C. Orellana‐Tavra , S. A. Mercado , and D. Fairen‐Jimenez , “Endocytosis Mechanism of Nano Metal‐Organic Frameworks for Drug Delivery,” Advanced Healthcare Materials 5 (2016): 2261–2270, 10.1002/adhm.201600296. 27385477

[17] E. Linnane , S. Haddad , F. Melle , Z. Mei , and D. Fairen‐Jimenez , “The Uptake of Metal–Organic Frameworks: A Journey Into the Cell,” Chemical Society Reviews 51 (2022): 6065–6086, 10.1039/D0CS01414A. 35770998 PMC9289890

[18] Y. H. Kiang , G. B. Gardner , S. Lee , Z. Xu , and E. B. Lobkovsky , “Variable Pore Size, Variable Chemical Functionality, and an Example of Reactivity Within Porous Phenylacetylene Silver Salts,” Journal of the American Chemical Society 121 (1999): 8204–8215, 10.1021/ja991100b.

[19] Z. Wang and S. M. Cohen , “Postsynthetic Covalent Modification of a Neutral Metal−Organic Framework,” Journal of the American Chemical Society 129 (2007): 12368–12369, 10.1021/ja074366o. 17880219

[20] P. Horcajada , T. Chalati , C. Serre , et al., “Porous Metal–Organic‐Framework Nanoscale Carriers as a Potential Platform for Drug Delivery and Imaging,” Nature Materials 9 (2009): 172–178, 10.1038/nmat2608. 20010827

[21] X. Chen , Y. Zhuang , N. Rampal , et al., “Formulation of Metal–Organic Framework‐Based Drug Carriers by Controlled Coordination of Methoxy PEG Phosphate: Boosting Colloidal Stability and Redispersibility,” Journal of the American Chemical Society 143 (2021): 13557–13572, 10.1021/jacs.1c03943. 34357768 PMC8414479

[22] S. Wang , W. Morris , Y. Liu , et al., “Surface‐Specific Functionalization of Nanoscale Metal–Organic Frameworks,” Angewandte Chemie International Edition 54 (2015): 14738–14742, 10.1002/anie.201506888. 26492949

[23] S. Wuttke , S. Braig , T. Preiß , et al., “MOF Nanoparticles Coated by Lipid Bilayers and Their Uptake by Cancer Cells,” Chemical Communications 51 (2015): 15752–15755, 10.1039/C5CC06767G. 26359316

[24] X. Liu , J. Obacz , G. Emanuelli , et al., “Enhancing Drug Delivery Efficacy Through Bilayer Coating of Zirconium‐Based Metal–Organic Frameworks: Sustained Release and Improved Chemical Stability and Cellular Uptake for Cancer Therapy,” Chemistry of Materials 36 (2024): 3588–3603, 10.1021/acs.chemmater.3c02954. 38681089 PMC11044268

[25] K. S. Butler , C. J. Pearce , E. A. Nail , G. A. Vincent , and D. F. Sava Gallis , “Antibody Targeted Metal–Organic Frameworks for Bioimaging Applications,” ACS Applied Materials & Interfaces 12 (2020): 31217–31224, 10.1021/acsami.0c07835. 32559362

[26] K. Alt , F. Carraro , E. Jap , et al., “Self‐Assembly of Oriented Antibody‐Decorated Metal–Organic Framework Nanocrystals for Active‐Targeting Applications,” Advanced Materials 34 (2022): 2106607, 10.1002/adma.202106607. 34866253

[27] W. Morris , W. E. Briley , E. Auyeung , M. D. Cabezas , and C. A. Mirkin , “Nucleic Acid–Metal Organic Framework (MOF) Nanoparticle Conjugates,” Journal of the American Chemical Society 136 (2014): 7261–7264, 10.1021/ja503215w. 24818877

[28] J. S. Kahn , L. Freage , N. Enkin , M. A. A. Garcia , and I. Willner , “Stimuli‐Responsive DNA‐Functionalized Metal–Organic Frameworks (MOFs),” Advanced Materials 29 (2017): 1602782, 10.1002/adma.201602782. 27922207

[29] S. Wang , C. M. McGuirk , M. B. Ross , et al., “General and Direct Method for Preparing Oligonucleotide‐Functionalized Metal–Organic Framework Nanoparticles,” Journal of the American Chemical Society 139 (2017): 9827–9830, 10.1021/jacs.7b05633. 28718644 PMC5572147

[30] W. H. Chen , S. Yang Sung , M. Fadeev , A. Cecconello , R. Nechushtai , and I. Willner , “Targeted VEGF‐Triggered Release of an Anti‐Cancer Drug From Aptamer‐Functionalized Metal–Organic Framework Nanoparticles,” Nanoscale 10 (2018): 4650–4657, 10.1039/C8NR00193F. 29465130

[31] X. Chen , S. M. Argandona , F. Melle , N. Rampal , and D. Fairen‐Jimenez , “Advances in Surface Functionalization of Next‐Generation Metal‐Organic Frameworks for Biomedical Applications: Design, Strategies, and Prospects,” Chemistry 10 (2024): 504–543, 10.1016/j.chempr.2023.09.016.

[32] I. Abánades Lázaro , S. Haddad , S. Sacca , C. Orellana‐Tavra , D. Fairen‐Jimenez , and R. S. Forgan , “Selective Surface PEGylation of UiO‐66 Nanoparticles for Enhanced Stability, Cell Uptake, and pH‐Responsive Drug Delivery,” Chemistry 2 (2017): 561–578, 10.1016/j.chempr.2017.02.005. PMC5421152 28516168

[33] E. Siouve , F. Melle , C. F. Martins , et al., “A General Strategy for Site‐Oriented Protein Anchoring on Metal–Organic Frameworks: HER2‐Targeted Delivery Using an Engineered Antibody Fragment,” ChemRxiv (2026), 10.26434/CHEMRXIV.15002077/V1.

[34] S. Wang , S. S. Park , C. T. Buru , et al., “Colloidal Crystal Engineering With Metal–Organic Framework Nanoparticles and DNA,” Nature Communications 11 (2020): 2495, 10.1038/s41467-020-16339-w. PMC7237412 32427872

[35] Q. Wang , L. Chen , Y. Long , H. Tian , and J. Wu , “Molecular Beacons of Xeno‐Nucleic Acid for Detecting Nucleic Acid,” Theranostics 3 (2013): 395–408, 10.7150/thno.5935. 23781286 PMC3677410

[36] B. Hu , L. Zhong , Y. Weng , et al., “Therapeutic siRNA: State of the Art,” Signal Transduction and Targeted Therapy 5 (2020): 101, 10.1038/s41392-020-0207-x. 32561705 PMC7305320

[37] P. E. Nielsen , M. Egholm , R. H. Berg , and O. Buchardt , “Sequence‐Selective Recognition of DNA by Strand Displacement With a Thymine‐Substituted Polyamide,” Science (1979) 254 (1991): 1497–1500, 10.1126/science.1962210. 1962210

[38] F. Pellestor and P. Paulasova , “The Peptide Nucleic Acids (PNAs), Powerful Tools for Molecular Genetics and Cytogenetics,” European Journal of Human Genetics 12 (2004): 694–700, 10.1038/sj.ejhg.5201226. 15213706

[39] P. E. Nielsen and M. Egholm , “An Introduction to Peptide Nucleic Acid,” Current Issues in Molecular Biology 1 (1999): 89–104, 10.21775/cimb.001.089. 11475704

[40] U. Giesen , W. Kleider , C. Berding , A. Geiger , H. Ørum , and P. E. Nielsen , “A Formula for Thermal Stability (Tm) Prediction of PNA/DNA Duplexes,” Nucleic Acids Research 26 (1998): 5004–5006, 10.1093/nar/26.21.5004. 9776766 PMC147916

[41] G. L. Wilson , B. S. Dean , G. Wang , and D. A. Dean , “Nuclear Import of Plasmid DNA in Digitonin‐Permeabilized Cells Requires Both Cytoplasmic Factors and Specific DNA Sequences,” Journal of Biological Chemistry 274 (1999): 22025–22032, 10.1074/jbc.274.31.22025. 10419528 PMC4397984

[42] R. Mejia‐Ariza , J. Rosselli , C. Breukers , et al., “DNA Detection by Flow Cytometry Using PNA‐Modified Metal–Organic Framework Particles,” Chemistry—A European Journal 23 (2017): 4180–4186, 10.1002/chem.201605803. 28139850 PMC5396136

[43] D. Feng , Z. Y. Gu , J. R. Li , H. L. Jiang , Z. Wei , and H. C. Zhou , “Zirconium‐Metalloporphyrin PCN‐222: Mesoporous Metal–Organic Frameworks With Ultrahigh Stability as Biomimetic Catalysts,” Angewandte Chemie International Edition 51 (2012): 10307–10310, 10.1002/anie.201204475. 22907870

[44] W. Morris , B. Volosskiy , S. Demir , et al., “Synthesis, Structure, and Metalation of Two New Highly Porous Zirconium Metal–Organic Frameworks,” Inorganic Chemistry 51 (2012): 6443–6445, 10.1021/ic300825s. 22676251

[45] J. W. M. Osterrieth , J. Rampersad , D. Madden , et al., “How Reproducible are Surface Areas Calculated From the BET Equation?,” Advanced Materials 34 (2022): 2201502, 10.1002/adma.202201502. 35603497

[46] P. Arenas‐Guerrero , Á. V. Delgado , K. J. Donovan , et al., “Determination of the Size Distribution of Non‐Spherical Nanoparticles by Electric Birefringence‐Based Methods,” Scientific Reports 8 (2018): 9502, 10.1038/s41598-018-27840-0. 29934624 PMC6015062

[47] H. DeVoe and I. Tinoco , “The Hypochromism of Helical Polynucleotides,” Journal of Molecular Biology 4 (1962): 518–527, 10.1016/S0022-2836(62)80106-5. 13885893

[48] F. G. Moscoso , J. J. Romero‐Guerrero , D. Rodriguez‐Lucena , J. M. Pedrosa , and C. Carrillo‐Carrión , “Nanosized Porphyrinic Metal–Organic Frameworks for the Construction of Transparent Membranes as a Multiresponsive Optical Gas Sensor,” Small Science 4 (2024): 2400210, 10.1002/smsc.202400210. 40212252 PMC11935058

[49] S. Cherian and C. C. Wamser , “Adsorption and Photoactivity of Tetra(4‐carboxyphenyl)Porphyrin (TCPP) on Nanoparticulate TiO2,” Journal of Physical Chemistry B 104 (2000): 3624–3629, 10.1021/jp994459v.

[50] R. R. Sinden , C. E. Pearson , V. N. Potaman , and D. W. Ussery , “DNA: Structure and Function,” Advances in Genome Biology 5 (1998): 1–141.

[51] A. Rusu and E. L. Buta , “The Development of Third‐Generation Tetracycline Antibiotics and New Perspectives,” Pharmaceutics 13 (2021): 2085, 10.3390/pharmaceutics13122085. 34959366 PMC8707899

[52] D. A. Talan , K. G. Naber , J. Palou , and D. Elkharrat , “Extended‐Release Ciprofloxacin (Cipro XR) for Treatment of Urinary Tract Infections,” International Journal of Antimicrobial Agents 23 (2004): 54–66, 10.1016/j.ijantimicag.2003.12.005. 15037329

[53] M. Cedrún‐Morales , M. Migliavacca , M. Ceballos , et al., “Clickable Polymer‐Based Coatings for Modulating the Interaction of Metal–Organic Framework Nanocrystals With Living Cells,” ACS Applied Materials & Interfaces 17 (2025): 24994–25010, 10.1021/acsami.5c01695. 40257304 PMC12131220

[54] W. H. Chen , X. Yu , A. Cecconello , Y. S. Sohn , R. Nechushtai , and I. Willner , “Stimuli‐Responsive Nucleic Acid‐Functionalized Metal–Organic Framework Nanoparticles Using pH‐ and Metal‐Ion‐Dependent DNAzymes as Locks,” Chemical Science 8 (2017): 5769–5780, 10.1039/c7sc01765k. 28989617 PMC5621505

[55] Y. Jiang , W. Zheng , K. Tran , et al., “Hydrophilic Nanoparticles That Kill Bacteria While Sparing Mammalian Cells Reveal the Antibiotic Role of Nanostructures,” Nature Communications 13 (2022): 197, 10.1038/s41467-021-27193-9. PMC8752835 35017467

[56] M. H. Zwietering , I. Jongenburger , F. M. Rombouts , and K. Van't Riet , “Modeling of the Bacterial Growth Curve,” Applied and Environmental Microbiology 56 (1990): 1875–1881, 10.1128/aem.56.6.1875-1881.1990. 16348228 PMC184525

[57] F. Ströhl and C. F. Kaminski , “A Joint Richardson—Lucy Deconvolution Algorithm for the Reconstruction of Multifocal Structured Illumination Microscopy Data,” Methods and Applications in Fluorescence 3 (2015): 014002, 10.1088/2050-6120/3/1/014002. 29148478

[58] L. J. Young , F. Ströhl , and C. F. Kaminski , “A Guide to Structured Illumination TIRF Microscopy at High Speed With Multiple Colors,” Journal of Visualized Experiments: JoVE 2016 (2016): e53988, 10.3791/53988. PMC4927749 27285848

[59] A. Markwirth , M. Lachetta , V. Mönkemöller , et al., “Video‐Rate Multi‐Color Structured Illumination Microscopy With Simultaneous Real‐Time Reconstruction,” Nature Communications 10 (2019): 4315, 10.1038/s41467-019-12165-x. PMC6754501 31541134

[60] C. Karras , M. Smedh , R. Förster , H. Deschout , J. Fernandez‐Rodriguez , and R. Heintzmann , “Successful Optimization of Reconstruction Parameters in Structured Illumination Microscopy—A Practical Guide,” Optics Communication 436 (2019): 69–75, 10.1016/j.optcom.2018.12.005.

[61] J. Sobczyński , H. H. Tønnesen , and S. Kristensen , “Influence of Aqueous Media Properties on Aggregation and Solubility of Four Structurally Related Meso‐Porphyrin Photosensitizers Evaluated by Spectrophotometric Measurements,” Die Pharmazie 68 (2013): 100–109, 10.31083/ph.2013.2130. 23469681

[62] H. R. Jiménez , M. Julve , and J. Faus , “A Solution Study of the Protonation and Deprotonation Equilibria of 5,10,15,20‐tetra(p‐sulphonatophenyl)Porphyrin. Stability Constants of Its Magnesium(II), Copper(II) and Zinc(II) Complexes,” Journal of the Chemical Society, Dalton Transactions 8 (1991): 1945–1949.

[63] M. Monsigny , C. Sene , A. Obrenovitch , A.‐C. Roche , F. Delmotte , and E. Boschetti , “Properties of Succinylated Wheat‐Germ Agglutinin,” European Journal of Biochemistry 98 (1979): 39–45, 10.1111/j.1432-1033.1979.tb13157.x. 467446

