# Supplementary material for: DNA‐mimic for Specific Surface Functionalization of Zr‐MOFs for Bacterial Targeting
Source: Angew Chem Int Ed Engl. 2026 May 23;65(30):e25762. doi: 10.1002/anie.202525762 (PMC13383184; doi:10.1002/anie.202525762)
Supplement: Supplementary file 1 — The authors have cited additional references within the Supporting Information [21, 29, 37, 40, 45, 56, 57, 58, 59, 60, 61, 62, 63]. Supporting File: anie72381‐sup‐0001‐SuppMat.docx. [file ANIE-65-e25762-s001.docx]

# **Supplementary information**

# **DNA-mimic for Specific Surface Functionalization of Zr-MOFs for Bacterial Targeting**

**Anna Scheeder****^1^***, Jon Ostolaza-Paraiso****^1^***, Andrew G. Baker** *^1^***, Juan F. Blandez** *^1^***, Georgina E. Lindop^2^, Dr. Simon M. Fairclough^2^, Prof. Ljiljana Fruk ^1^, Ioanna Mela#***^3^***, David Fairen‐Jimenez#***^1^***, Clemens F. Kaminski#***^1^*

*equal contribution

# Corresponding Authors

*^1^Department of Chemical Engineering & Biotechnology, University of Cambridge, Philippa Fawcett Drive, Cambridge CB3 0AS, United Kingdom*

*^2^Department of Material Science and Metallurgy, University of Cambridge, Charles Babbage Rd, Cambridge CB3 0FS*

*^3^Department of Pharmacology, University of Cambridge, Tennis Ct Rd, Cambridge CB2 1QR*

Table of Contents

[**S1 General experimental remarks** 2](#_Toc192060899)

[**S1.1 Materials** 2](#_Toc192060900)

[**S1.2 Physicochemical characterization of materials** 3](#_Toc192060901)

[**S2 General synthetic procedures** 6](#_Toc192060902)

[**S3 *In vitro* methods** 11](#_Toc192060903)

[**S4 Characterization of PCN-222** 15](#_Toc192060904)

[**S5 Additional strategies to promote specific Zr–O–P interactions** 18](#_Toc192060905)

[S5.1 Salt ageing 18](#_Toc192060906)

[S5.2 pH titration 19](#_Toc192060907)

[**S6 Characterization of NA-coated PCN-222** 20](#_Toc192060908)

[**S7 Characterization of WGA, Atto-bridge, and WGA-bridge** 24](#_Toc192060909)

[**S8 Assessment of drug-loaded PCN-222** 27](#_Toc192060910)

## **S1 General experimental remarks**

### **S1.1 Materials**

Sodium chloride, sodium hydroxide (>97%), hydrochloric acid (37%), calcium chloride dihydrate (>99%), ciprofloxacin hydrochloride, tetracycline hydrochloride, wheat germ agglutinin, TBE buffer, agarose, Tris-HCL, magnesium chloride, EZ-Link™ DBCO Protein Labeling Kit, glycerol, EDTA, MES, Tris base, SDS, DTT, bromophenol blue were purchased from Sigma Aldrich. FITC-labelled wheat germ agglutinin and chitin hydrolysate were purchased from 2BScientific. InstantBlue® Coomassie Protein Stain was acquired from Abcam.

Phosphate buffer saline and lysogeny broth (LB) media were provided by the Department of Chemical Engineering and Biotechnology. Origami buffer was synthesized from 2 mM MgCl2 and 10 mM Tris (pH 7.6) The NuPAGE™ Bis-Tris Mini Protein Gels, Spectra™ Multicolor Broad Range Protein Ladder and the dyes MitoTracker™ Orange, SYTO™ 9, SynaptoGreen™, SynaptoRed™ C2, and MitoTracker™ Red were all purchased from ThermoFisher Scientific.

All DNA sequences and nuclease free water were purchased from Integrated DNA Technologies. The PNA sequence was purchased from LifeTein and the information about the sequences is found in Table 1.

**Table S1.1: NA Sequences for PCN-222 Surface Modifications**

| **Name** | **Sequence (5’ to 3’)** | **Number of Bases** |
| --- | --- | --- |
| DNA | GAG CTA CCA CTT CAC TCC AGT TTT TTT TT | 20+9T |
| DNA_Phos_ | GAG CTA CCA CTT CAC TCC AGT TTT TTT TT/3Phos/ | 20+9T |
| PNA_Phos_ | CCA CTT CAC TCC AGT TTT TT-{pTyr}-NH2 | 14+6T |

**Table S1.2: NA Sequences for DNA-based Targeting Bridge.** Bold, italic and underlined sequences have complementary nucleotides.

| **Name** | **Sequence (5’ to 3’)** | **Number of Bases** |
| --- | --- | --- |
| B1 | **AGT CTA TCA GTC CGG ATT CG**C GTC TGC GGC TAT TGC TCG A | 40 |
| B2 | *CTG GAG TGA AGT GGT AGC TC*T CGA GCA ATA GCC GCA GAC G | 40 |
| B2-PNA | *CTG GAG TGA AGT GGT CGA GCA ATA GCC GCA GAC G* | 34 |
| ssDNA-Atto561 | **CGA ATC CGG ACT GAT AGA CT**T TTT /3ATTO565N/ | 20+4T |
| ssDNA-Azide | **CGA ATC CGG ACT GAT AGA CT**T TTT/3AzideN/ | 20+4T |
| ssDNA-FAM | *GAG CTA CCA CTT CAC TCC AG*T T/36-FAM/ | 20+2T |
| PNA_Phos_ | *CCA CTT CAC TCC AGT TTT TT-{pTyr}-NH2* | 14+6T |

### **S1.2 Physicochemical characterization of materials**

**Powder X-ray diffraction (PXRD)**: Experimental diffraction patterns were collected at room temperature with a Bruker D8 Advance diffractometer with a Cu Kα1 radiation (λ=1.5406 Å) on a mounted bracket sample stage (2θ = 2-30º). The samples were first dried in the oven and were then ground to obtain powders. The experimental PXRD patterns were compared to the theoretical or calculated PXRD patterns of the MOFs, which were obtained with Mercury by using the corresponding cif files. The cif files were downloaded from the Cambridge Structural Database (CSD), a unit of the Cambridge Crystallographic Data Centre (CCDC) that acts as a repository for crystal structures.

**Dynamic light scattering (DLS)**: The particle size in solution after the formation of the coating was checked by DLS. The measurements were carried out at 25 ºC using a Zetasizer Nano ZS (Malvern Instrument Ltd., UK) equipped with a He-Ne laser operating at 633 nm. A solution of 0.1 mg/ml of the corresponding material was transferred to a disposable cuvette, where three measurements of at least 11 runs of ten seconds each were carried out to increase its accuracy. The standard deviation among the three measurements is represented with error bars.

**Adsorption of N_2_**: The surface area and the pore volume of PCN-222 was measured by adsorption of N_2_ at 77 K. For the successful measurement of the samples, at least 30 mg of dried material were first activated at 120 ºC overnight under vacuum in a vacuum oven (OV-12, Lab Companions Shop), followed by the further activation of the materials in a Micromeritics VacPrep degasser at 120 ºC for 8 hours. The tubes were filled with glass rods to reduce the empty volume and were surrounded by isothermal jackets, thus maintaining a uniform temperature along the whole sample tube. The temperature of the system was controlled by adding a dewar filled with liquid N_2_. The analysis of the textural properties of the MOFs was carried out with BET surface identification (BETSI), a software tool developed by Osterrieth *et al*.^[43]^

**Scanning electron microscopy (SEM)**: The surface topology and the size of the nanoparticles after being coated was checked by SEM. To prepare the samples for the correct acquisition of the images, a small piece of conductive Cu tape was attached to the SEM stub. Then, a previously sonicated solution of the sample in ethanol was drop casted into the tape, instantly evaporating the solvent. Before the measurement, the samples were coated for 30 seconds with a thin conductive layer of Pt using a Polaron SC7640 sputter coater. Images were taking using a FEI Nova NanoSEM, using a spot size of 2.5 and an acceleration voltage of 5 kV. The particle size distribution of the sample was assessed manually with the software ImageJ, by measuring the size of 50-100 individual nanoparticles.

**Transmission electron microscopy (TEM)**: The crystallinity and size of the samples was also assessed by TEM. An aqueous solution of the nanoparticles was first sonicated and drop casted into carbon-coated 200 mesh copper grids (Science Services). The grids were then fitted into a single-tilt sample holder. TEM and STEM images were acquired using a Talos F200X G2 with an acceleration voltage of 200 keV.

**Atomic Force Microscopy (AFM)**: The surface features of PCN-222, DNA@PCN-222, DNA_Phos_@PCN-222, and PNA_Phos_@PCN-222 were characterized using AFM. A 10 μl aliquot of MOF suspension (50 μg/ml in deionized water) was deposited onto a spermidine-treated mica sheet. The mica sheet was prepared by cleaving freshly, incubating with 20 μl of 5 mg/ml spermidine for 5 minutes, rinsing thoroughly with deionized water, and drying under a nitrogen flow. After deposition, the samples were allowed to dry at room temperature until the water had completely evaporated. AFM images of the samples were acquired using a Dimension FastScan® atomic force microscope (Bruker) in FastScan Bio mode. FASTSCAN-A probes (Bruker) with a nominal spring constant of 18 N/m and a resonant frequency of 1400 kHz were employed. AFM topography and phase images were recorded at scan speeds of 30 Hz. The recorded data were processed and first-order flattened using Nanoscope analysis software 2.0.

**Zeta potential**: The surface charge of the nanoparticles was assessed by zeta potential. The measurements were carried out at 25 ºC using a Zetasizer Nano ZS (Malvern Instrument Ltd., UK) equipped with a He-Ne laser operating at 633 nm. A solution of 0.1 mg/ml of the corresponding material was transferred to a disposable folded capillary cell (Malvern), where three measurements of at least 20 runs each were carried out to increase its accuracy. The standard deviation among the three measurements is represented with error bars.

## **S2 General synthetic procedures**

**Synthesis of PCN-222**: PCN-222 was synthesized following a previously published procedure from Chen *et al*. with slight modifications.^[22]^ First, 22.5 µg (14 µmol) of H_2_TCPP was added to 8 ml DMF. The solution was sonicated briefly until the solid was completely dissolved. Then, 38 mg (28 µmol) of Zr₆O_8_ clusters were added to the solution. Once fully dissolved, 200 µl of trifluoroacetic acid (TFA), were added. The solution was then transferred to a previously heated aluminum block at 120 ºC and was stirred at 400 rpm for five hours. After reaction, the vials were cooled down to room temperature, and they were washed three times with DMF to remove any unreacted chemicals or by products, followed by solvent exchange with ethanol three times to remove any residual DMF. Finally, the product was dispersed in ethanol and stored at 4 ºC.

**Synthesis of DNA@PCN-222, DNA_Phos_@PCN-222, PNA_Phos_@PCN-222**: First, previously synthesized PCN-222 (1,000 µg/ml) in ethanol was solvent exchanged twice with DI water via centrifugation and resuspension (30 minutes, 14,850 rpm). In the meantime, working solutions of 30 ng/µl of DNA, DNA_Phos_, and PNA_Phos_ in nuclease free water were made and the initial concentrations of the working solutions were obtained via UV-Vis measurements with the NanoDrop. Then, 900 µl of each solution was added to 100 µl of PCN-‑222 in water, for a total volume of 1,000 µl. The final solutions were incubated overnight on a rotary shaker at RT (20 ºC). The morning after, the samples were washed three times with DI water at 2,400g for 10 min and the final coated materials were resuspended in 1,000 µl of DI water. The supernatant of the first wash was used to quantify the remaining unbound ssDNA or PNA via UV-Vis measurement with the NanoDrop. The amount attached to the MOF was quantified after the last washing step. The MOF was finally stored at 4 ºC for further use.

**Synthesis of O-Phospho-L-tyrosine@PCN-222:** First, previously synthesized PCN-222 (1,000 µg/ml) in ethanol was solvent exchanged twice with DI water via centrifugation and resuspension (30 minutes, 14,850 rpm). In the meantime, working solutions of 0.1 and 1 ng/µl of the O-Phospho-L-tyrosine (Sigma-Aldrich) in nuclease free water were made. Then, 900 µl of each solution was added to 100 µl of PCN-222 in water, for a total volume of 1,000 µl. The final solutions were incubated overnight on a rotary shaker at RT (20 ºC). The morning after, the samples were washed three times with DI water at 14,850 rpm for 10 minutes and the final coated materials were resuspended in 1,000 µl of DI water. The MOF was finally stored at 4 ºC for EM measurements.

**WGA conjugation to N_3_-modified ssDNA**: The protein conjugation to an azide‑modified ssDNA strand (CGA ATC CGG ACT GAT AGA CTT TTT /3AzideN/) was performed with an EZ-Link™ DBCO Protein Labelling Kit according to the seller’s instructions. Briefly, the WGA protein was DBCO-labelled by mixing 16 μl of the DMSO reconstituted EZ-linker with 100 μl of WGA solution (1 mg/ml in 1x PBS). The reaction mixture was incubated for 1 hour at room temperature using a rotary shaker. Next, the DBCO-labelled protein was purified using a freshly prepared Zeba™ Dye and Biotin Removal Spin Column through centrifugation at 1,000g for 2 minutes. The DBCO‑modified protein was immediately used for conjugation to the N_3_-modified ssDNA or stored at 4 ºC for further use. If not otherwise mentioned, the ssDNA was added in a nine-molar excess to the protein by mixing 20 µl of ssDNA (100 µM in nuclease free water) with 20 µl protein conjugate at 37 ºC for 12 h using a rotary shaker. The protein conjugation was confirmed with a NuPAGE™ Bis-Tris Mini Protein Gels run at a 12% polyacrylamide concentration. No further purification was performed. The ssDNA‑WGA modification was finally stored at 4 ºC for further use.

**SDS-PAGE**: Protein samples were prepared by mixing 2 µl of home-made 6x sodium dodecyl sulphate (SDS) loading buffer (0.23 M Tris-HCl pH 6.8, 24% v/v glycerol, 120 µM bromophenol blue, 0.4 M DTT, 0.23 M SDS, stored at -20 °C) with 10 µl of each sample, followed by boiling at 90 °C for 10 minutes for denaturation. NuPAGE Novex 4-12% Bis-Tris gels were used, set up with ice-cold home-made MES running buffer (50 mM MES, 50 mM Tris base, 1 mM EDTA, 0.1% w/v SDS, pH 7.3) in the inner chamber. Each gel well was loaded with 10 µl of the denatured samples, along with 5 µl of Spectra™ Multicolor Broad Range Protein Ladder as a molecular weight marker. Electrophoresis was performed at a constant voltage of 180 V for 50 minutes. Post‑electrophoresis, gels were stained using InstantBlue® Coomassie Protein Stain for 15 minutes, followed by washing out residual dye in water for 20 minutes. Gels were imaged with a Genesys GBOX system.

**Atto-bridge assembly**: The Atto561 dye-containing DNA bridge, referred to as Atto‑bridge, was synthesized by adding 200 µl of each of B1, B2, and ssDNA-Atto561 at a working solution of 1 µM in 1x OB supplemented with 1 mM CaCl_2_. The final solution was mixed using a rotary shaker at 37 ºC for 30 min, with the mixture covered with aluminum foil to avoid light-induced degradation. The assembly of the bridge was checked by gel electrophoresis.

**Agarose-gel electrophoresis**: Agarose gel was used to check the successful hybridization of B1, B2 and ssDNA-Atto561. A 40 ml solution of 2.5 % agarose gel (1 mg in 40 ml) in 0.5x TBE buffer at pH 8.3 was prepared. 5 µl of the ladder (1 kb) and ~100 ng of the Atto-bridge were loaded and the gel was run for 45 min at 100 V. Gels were imaged with a Genesys GBOX system.

**WGA-bridge-Atto561 assembly for bacterial targeting conformation**: 200 µl of the four parts of the WGA-bridge-Atto561 (B1, B2, ssDNA-Atto561, and ssDNA-WGA) were added at a working solution (WS) of 1 µM in 1x OB supplemented with 1 mM CaCl_2_. Negative controls with only the ssDNA-WGA, without B2, or only the ssDNA‑Atto561 were also prepared. The final solution was mixed using a rotary shaker at 37 ºC for 30 min. The solution was covered with aluminum foil to avoid degradation by light. The assembly of the bridge was checked by gel electrophoresis. No further purification was performed.

**Atto-bridge assembly on NA-coated PCN-222**: First, a 500 nM WS of B1, B2 and ssDNA-Atto561 was prepared in 1x OB (1 µl each) to synthesize the Atto-bridge. Negative controls were prepared by adding ssDNA‑Atto561 only, ssDNA-Atto561 and B1, and ssDNA-Atto561and B2. The final solution was incubated for 30 min at 37 ºC. Then, 50 µl of DNA/DNA_Phos_/PNA_Phos_‑coated PCN-‑222 in water (100 µg/ml) were mixed with 50 µl of the Atto-bridge and incubated further for 30 minutes at 37 ºC. The samples were washed via centrifugation (2,400g for 10 min) and resuspension three times in 50 µl of the targeting buffer (1x PBS supplemented with 1 mM CaCl_2_).

**WGA-bridge assembly on NA-coated PCN-222**: First, 200 µl of a 500 nM WS of B1, B2, and ssDNA-WGA was prepared in 1x OB supplemented with 1 mM CaCl_2_ (1 µl each) to synthesize the WGA-bridge. A negative control with only the ssDNA‑WGA was also prepared. The final solution was incubated for 30 min at 37 ºC. Then, 50 µl of DNA/DNA_Phos_/PNA_Phos_-coated PCN-222 in water (100 µg/ml) were mixed with 50 µl of the WGA-bridge and incubated further for 30 minutes at 37 ºC. The samples were washed via centrifugation (2,400g for 10 min) and resuspension three times in 50 µl of the targeting buffer (1x PBS supplemented with 1 mM CaCl_2_). The samples were labelled as WGA@PCN-222, WGA@DNA@PCN-222, WGA@DNA_Phos_@PCN-222, and WGA@PNA_Phos_@PCN-‑222, depending on the coating of PCN-222.

**pH titration of PCN-222**: First, PCN-222 in ethanol was solvent exchanged twice with DI water via centrifugation and resuspension, obtaining a stock solution of PCN-222 in water at a concentration of 1,000 µg/ml. Then, eight different solutions were prepared by adding 200 µl of the stock solution and further diluting them in 1,800 µl of DI water, obtaining a total volume of 2,000 µl. Then, 1 M stock solutions of NaOH and HCl in water were made and gradual amounts of the stock solutions were added to the different aliquots to obtain solutions of PCN-222 in water at pH values that go from 3 to 10. The pH was determined using pH paper. The experiments were done in triplicate. The solutions were then photographed, and their stability was checked with DLS and zeta potential measurements.

**Salt ageing of DNA-coated PCN-222**: First, a 0.5 ml solution of PCN-222 in DI water (200 µg/ml) was brought to pH 8, upon which 40 µl of either DNA or DNA_Phos_ (100 µM) were added and incubated for 2 h. Then, 20 µl and 500 µl of 0.5 M NaCl were added to the MOF suspension, and DI water was added to have a final reaction volume of 1 ml (final NaCl concentrations were 10 and 250 mM). The pH of the reaction was adjusted back to 8, and the suspension was incubated overnight at room temperature using a rotary shaker. The following day, the MOFs were washed twice with DI water, and the supernatant of the first wash and the final material was analyzed with NanoDrop spectrophotometry.

**Single drug loading of ciprofloxacin and tetracycline in PCN-222**: Ciprofloxacin and tetracycline were encapsulated in PCN-222 using the following procedure: First, 2 mg/ml stock solutions of ciprofloxacin in water and tetracycline in ethanol were prepared. The solvents were chosen due to the high solubility of ciprofloxacin in water (30 mg/ml) and tetracycline in ethanol (~10-20 mg/ml). The initial concentration of the stock solutions were obtained by measuring the absorbance of ciprofloxacin and tetracycline at 277 and 366 nm, respectively, in water and ethanol, and back‑calculated using a previously made calibration curve in the corresponding solvent. Then, 1 mg/ml stock solutions of PCN-222 in water and ethanol were prepared. For the aqueous stock solution, PCN-222 in ethanol was solvent exchanged with water twice via centrifugation and resuspension. Once PCN-222 was resuspended in the desired solvent, 1 ml of the stock solution was transferred to a 4 ml glass vial. Then, 1 ml of the drug stock solution was added to the PCN-222 solution with the same solvent, obtaining a total reaction volume of 2 ml. The solution was then stirred at room temperature for 72 h, and the glass vial was covered with aluminum foil to avoid the degradation of the drugs by the light. After three days, the solutions were centrifuged (30 minutes, 14,850g) and the supernatant was collected. The absorbance of the supernatant was then measured at 277 and 366 nm in water and ethanol, respectively, to determine the amount of unloaded drug left in solution. The drug-loaded PCN-222 solutions were washed twice more, and the tetracycline-loaded PCN-222 was solvent exchanged with water twice via centrifugation and resuspension. The final drug-loaded materials (1 mg/ml of PCN-222 in water) were stored in the fridge at 4 ºC for further use. After drug encapsulation, the single drug-loaded PCN-222 were coated with PNA_Phos_ as explained in 5.2.3 to obtain PNA_Phos_@Ci@PCN-222 and PNA_Phos_@Te@PCN-222. The following nomenclature is used throughout the rest of this chapter. The drug loading was calculated using the equation described following:


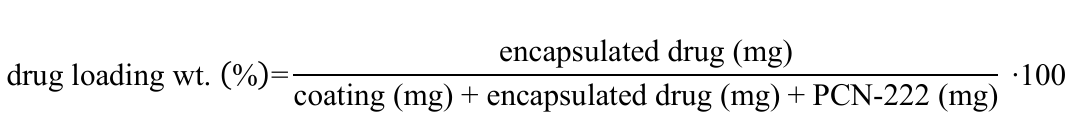


**Drug release of ciprofloxacin in PBS**

The release of ciprofloxacin from coated (PNA_Phos_@Ci@PCN-222) and uncoated (Ci@PCN-222) materials was studied in PBS by means of UV-Vis. First, ten aliquots with 900 μl of PBS were prepared for each material, each aliquot corresponding to a different time point (0 h, 0.5 h, 1 h, 2 h, 4 h, 8 h, 24 h, 36 h, 48 h, and 168 h). Then, 1 mg/ml solutions of PNA_Phos_@Ci@PCN-222 and Ci@PCN-222 were prepared in water. To carry out the experiments in PBS, the samples were solvent exchanged by centrifugation (14,850 rpm, 30 minutes) and resuspension. Finally, 100 μl of each nanoparticle solution were added to the previously prepared aliquot to obtain a final nanoparticle concentration of 100 μg/ml. The samples were then left in a rotary shaker for their corresponding time, upon which they were centrifuged (14,850 rpm, 30 minutes), and their supernatant collected. The absorbance of the supernatant was measured at 277 nm, which corresponds to the peak of ciprofloxacin. The amount of drug that got released into the solution was then calculated by comparing it to a previously made calibration curve in PBS.

## **S3 *In vitro* methods**

**Bacteria culture**: 10 mL of LB medium were inoculated with either *E. coli* MG1655 or BL21 (DE3) (Invitrogen) cells and incubated overnight at 37 °C in a shaking incubator.

**Biocompatibility studies of WGA:** Overnight cultures of MG1655 bacteria were diluted into fresh LB medium supplemented with 1 mM CaCl2 to an OD of ~0.025 (16 µl of culture into 10 mL of fresh LB). WGA was diluted into lectin binding buffer (1x PBS with 1 mM CaCl_2_) and 20 μl of the suspension was added to 180 μl of bacterial culture in each well to reach testing concentrations of 0, 1, 10, 100, and 1000 nM WGA in the final suspension. Each sample was tested in triplicate. The bacteria were grown over 16 hours in a shaking plate reader, at 37 °C, with OD600 measurements taken every 5 minutes. To extract growth rates, the recorded growth curves were fitted with a re‑parameterized Gompertz growth model in Origin 2019.^[53]^

**Bacterial targeting and glycan inhibition studies of FITC-WGA**: The starter cultures were diluted 100-fold in fresh LB medium and further grown until reaching an OD600 of 0.5. The culture was then washed three times through centrifugation (2,400g, 2 min) and resuspension in 1x PBS supplemented with 1 mM CaCl_2_. The *E. coli* cells were fluorescently labelled at 647 nm by membrane staining with MitoTracker™ Deep Red FM (Thermo Fisher). Therefore, 1 μM MitoTracker™ Deep Red was added to 1 ml of bacterial suspension for 15 minutes in a shaking incubator at 37 ºC. The cells were washed three times as previously described. Next, FITC-WGA was added to reach a final concentration of 1 μM (172.8 µl of the bacteria were mixed with 7.2 μl WGA (27.8 µM stock concentration) and 20 µl of buffer). A negative control with the inhibitory glycan chitin hydrolysate was prepared through the addition of 20 μl of the seller’s chitin hydrolysate solution (172.8 μl of the bacteria were mixed with 7.2 μl WGA (27.8 μM stock concentration) and 20 µl of chitin hydrolysate). The cells were incubated with the lectin solutions in the presence or absence of the glycan inhibitors for 15 min at 37 ºC. The cells were washed once through centrifugation at 2,400g for 2 minutes and resuspension in 1x PBS with 1 mM CaCl_2_ and imaged using a structured illumination microscope as described below.

**WGA bacterial targeting in co-culture experiments**: The overnight cultures of MG1655 and BL21 cells were diluted 100-fold in fresh LB medium and further grown until reaching an OD600 of 0.5. The cultures were then washed three times through centrifugation (2,400g, 2 min) and resuspension in 1x PBS supplemented with 1 mM CaCl_2_. The BL21 cells were fluorescently live labelled at 488 nm excitation with the cytoplasmic SYTO™ 9 Green Fluorescent Nucleic Acid Stain. The freshly washed cells were incubated with 5 μM SYTO™ 9 for 15 minutes in a shaking incubator at 37 ºC. The BL21 cells were washed three times as previously described before being used for further experiments, while MG1655 cells remained unstained. For WGA targeting studies, the stained BL21 and unstained MG1655 cells were mixed at a 1:1 ratio and incubated with 1 μM FITC-WGA in 1x PBS supplemented with 1 mM CaCl_2_ for 15 minutes. The cells were washed once through centrifugation at 2,400g for 2 minutes and resuspension in 1x PBS with 1 mM CaCl_2_ and imaged using a structured illumination microscope as described below.

**Biocompatibility studies of PCN-222, free drugs, and drug-loaded PCN-222 with MG1655 bacteria**: Overnight cultures of MG1655 bacteria were diluted into fresh LB medium supplemented with 1 mM CaCl_2_ to an OD of ~0.025. DNA-coated PCN-222 samples and the free drugs were diluted into lectin binding buffer (1x PBS with 1 mM CaCl_2_) and 20 μl of the suspension was added to 180 μl of bacterial culture in each well to reach testing concentrations of 0, 0.001, 1, and 10 μg/ml DNA-coated PCN-‑222; 1, 0.1, 0.01, 0.001 μg/ml ciprofloxacin and ciprofloxacin-loaded DNA-coated PCN-‑222; 10, 1, 0.1, 0.01 μg/ml tetracycline and tetracycline-loaded DNA-coated PCN-‑222; and 1 μg/ml tetracycline-loaded PNA-coated PCN-222 with bridge assembly in the final suspension. Each sample was tested in triplicates. The bacteria were grown over 16 hours in a shaking plate reader, at 37 °C, with OD600 measurements taken every 5 minutes. To extract growth rates, the recorded growth curves were fitted with a re-parameterized Gompertz growth model in Origin 2019.^[53]^

**PCN-222 bacterial targeting experiments**: WGA@DNAcoating@PCN-222 samples were prepared as previously described in section 5.2.6. Overnight cultures of MG1655 and BL21 cells were diluted 100-fold in fresh LB medium and further grown until reaching an OD600 of 0.5. The cultures were then washed three times through centrifugation (2,400g, 2 min) and resuspension in 1x PBS supplemented with 1 mM CaCl_2_. MG1655 cells were fluorescently live labelled at 488 nm excitation with the cytoplasmic SYTO™ 9 Green Fluorescent Nucleic Acid Stain while BL21 cells were stained with 1 μl MitoTracker™ Orange per 1 ml of bacterial suspension. For MG1655 targeting experiments, 50 µl of the MOF suspension were mixed with 50 µl of the fluorescently labelled bacteria resuspended in PBS supplemented with 1 mM CaCl_2_ for 15 minutes at 37 °C. In co-culture experiments, BL21 and MG1655 bacteria were mixed in 1:1 ratio and 50 µl of the MOF suspension were mixed with 50 µl of the fluorescently labelled bacteria resuspended in PBS supplemented with 1 mM CaCl_2_ for 15 minutes at 37 °C. The cells were prepared and imaged using a structured illumination microscope as described below.

**Mammalian Cell Culture:** Wi-38 (Catalog. No. CCL-75) a lung fibroblast cell line was purchased from American Type Culture Collection (ATCC). This cell line was maintained in DMEM (Sigma) supplemented with 10% FBS, 2 mM l-Glutamine and 1 mM sodium pyruvate (Sigma). All cell lines were incubated in 20% O_2_ and 5% CO_2_ at 37 °C and cultured in 0.25% Penicillin-Streptomycin (Gibco). Cells were routinely tested for mycoplasma using the universal Mycoplasma Detection Kit (ATCC) or by RNA‐capture ELISA.

**Viability Assays:** Cell viability was determined using CellTiter-Blue assay (Promega). Cells were seeded in 96-well plates (Eppendorf) at 5,000 cells/well. After 24 h, PCN-222, PCN-222 modified with DNA, PCN-222 modified with DNAPhos, and PCN-222 modified with PNA, were added to the cells for 72 h at concentrations of 100 μg/mL and 10 μg/mL for each condition. After 72 h, media was exchanged, and 4 μL CellTiter-Blue reagent were added to each well. After incubation for 2 h the absorbance and fluorescence were recorded for each well on the Infinite 200 Pro (Tecan) using 560/590 excitation emission. The viability studies were conducted in 3 technical replicates and 3 biological replicates. The viability was calculated according to the following equation: 100 x ((Sample – Media Control)/(Untreated Cells – Media Control)) as percent viability.

**Structured illumination microscopy**: 1 µl of MOF or bacteria samples were deposited onto a glass slide (Academy, 22x40 mm, 0.16-0.19 mm thick) and immobilized underneath a custom-made 5 x 5 mm agarose pad (1% w/v). The agarose pad was covered with a glass coverslip to prevent sample drying before imaging. Fluorescence microscopy images were acquired using a 3-colour SIM technique.^[54]^ A 60x 1.2 NA water immersion lens (UPLSAPO 60XW, Olympus) focused the structured illumination pattern onto the sample and captured the emitted fluorescence light, which was then projected onto an sCMOS camera (Orca-flash 4.0, Hamamatsu). Excitation wavelengths of 488 nm (iBEAM-SMART-488, Toptica), 561 nm (OBIS561, Coherent), and 640 nm (MLD 640, Cobolt) were used. Image acquisition was performed with custom SIM software,^[55]^ and reconstructions were carried out using the open-source software FairSIM,^[56]^ following best practices for parameter selection.^[57]^ Each pixel measured 107 nm during acquisition, corresponding to 53.5 nm after reconstruction. Experiments were conducted in a temperature-controlled room at 20 °C.

**Co-localization analysis**: A Spearman co-localization analysis was performed for FITC-WGA targeted bacteria and Atto-561 labelled MOF samples. To quantify and visualize co-localization between the two channels, the Spearman rank correlation coefficient (SPCC) was used. After Otsu thresholding each channel, the SPCC was calculated as


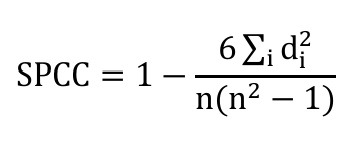


where 𝑛 is the number of pixels of the image and d describes the difference in rank (ranked regarding pixel intensity) of the two channels for each pixel 𝑖.

**Data visualization and statistical analysis**: Graphs were plotted using Origin 2019. Statistical significance between two values was assessed using a two-tailed, unpaired Student’s t-test (Origin 2019). Asterisks indicate statistical significance as determined by the Student’s t-test (*P < 0.05, **P < 0.01, ***P < 0.001, and ****P < 0.0001).

## **S4 Characterization of PCN-222**

**Figure S1: Physicochemical characterization of PCN-222.** **a**. PXRD patterns of simulated (black) and as-synthesized PCN-222 (purple). **b**. N_2_ adsorption isotherm of PCN-222 at 77 K. **c**. Particle size distribution of PCN-222 in water, obtained by DLS. **d**. Zeta potential of PCN-222, measured in water 1 and 24 h after solvent exchange. **e**. HAADF-STEM image of PCN-222. **f**. SEM image of PCN-222. Scale bars correspond to 100 nm.


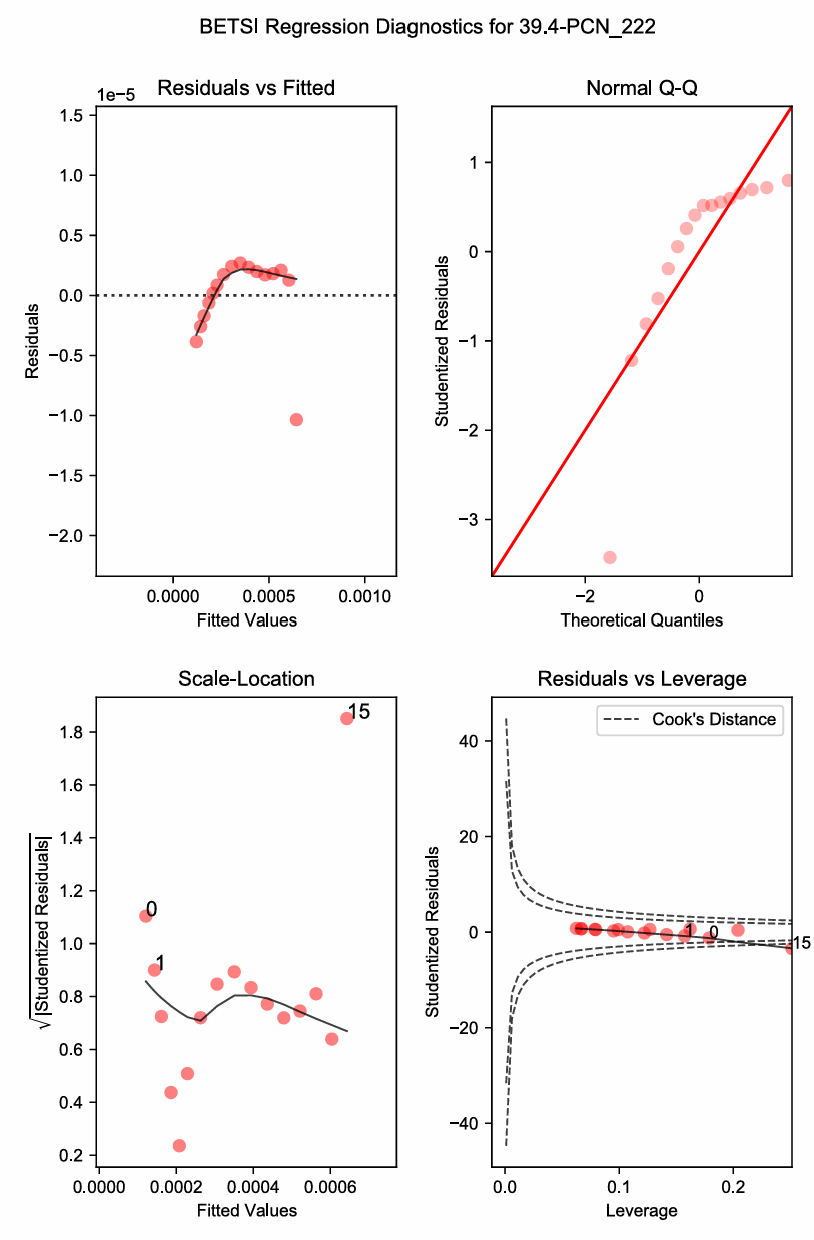


**Figure S2: BETSI regression diagnostics for PCN-222.**


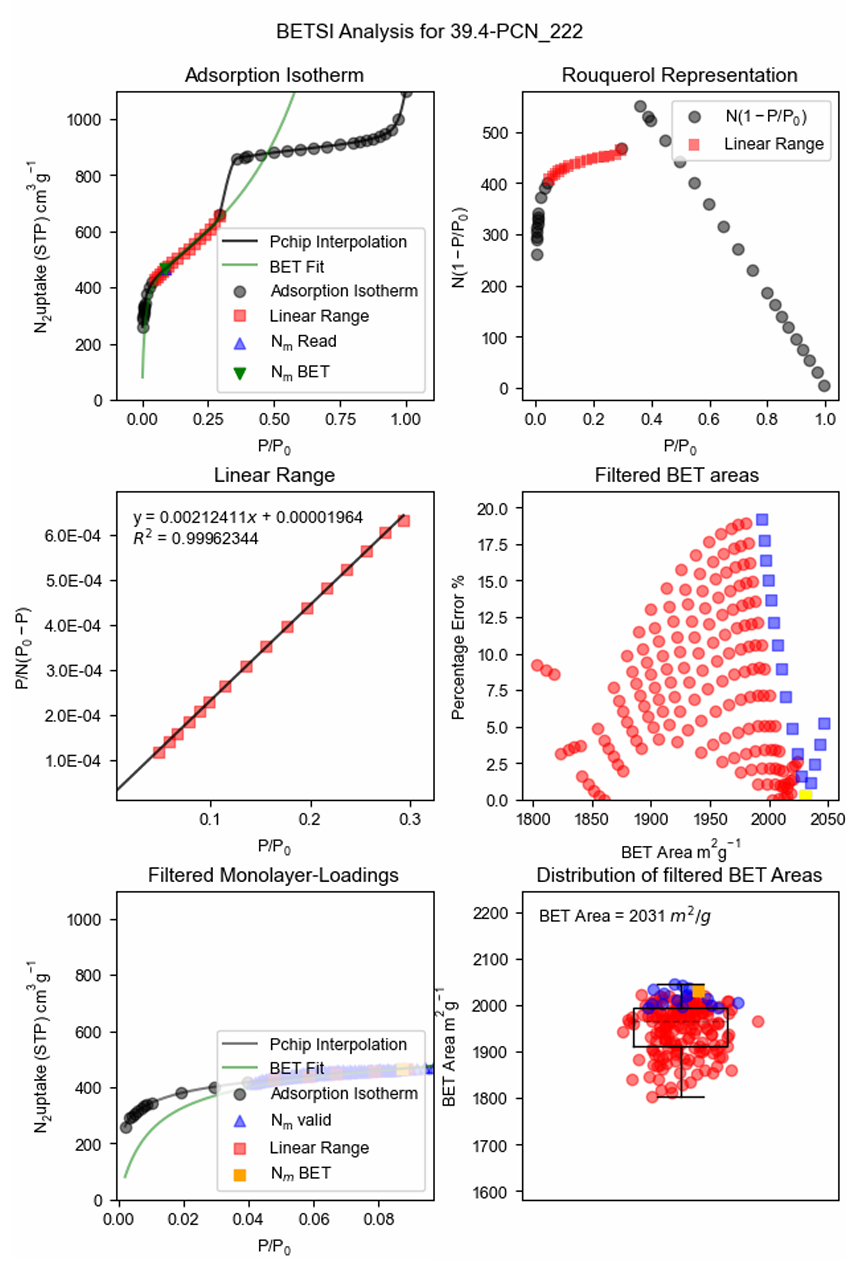


**Figure S3: BETSI analysis for PCN-222**.

## **S5 Additional strategies to promote specific Zr–O–P interactions**

### **S5.1 Salt ageing**

The coating process of PCN-222 did not exhibit specificity toward the phosphate terminal group present in DNA_Phos_. Like DNA_Phos_, non-modified DNA attached to the PCN-222 surface (**Fig. 2**). This suggests that electrostatic interactions between the negatively charged phosphate backbone of DNA and the positively charged Zr atoms facilitate surface attachment. As a result, DNA likely remains flat on the surface of the MOF rather than protruding outward due to the competition between the terminal phosphate and the phosphodiester backbone of the DNA, hindering its further hybridization with the targeting moiety.

To shield the effect of unspecific charge driven attachment of DNA with its backbone to PCN-222, Wang et al. suggested the use of a salt aging procedure that could stabilize the negatively charged oligomers.^[30]^ PCN-222 was therefore incubated with 4 μM DNA and DNA_Phos_ and the effect of the addition of salts was studied at two different NaCl concentrations, 10 mM and 250 mM. Figure S4 shows the particle size distribution and change in zeta potential of DNA and DNA_Phos_-coated PCN-222 in the presence of 10 and 250 mM NaCl.

The addition of NaCl in solution had varying effects in the stability of PCN-222. PCN-‑222 showed good colloidal stability in the presence of 10 mM NaCl, but significantly aggregated when the salt solution was increased to 250 mM, obtaining an average size of over 1000 nm, and a PDI of around 0.7. In contrast, DNA- and DNA_Phos_-coated PCN-222 exhibited great colloidal stability, suggesting that the addition of DNA stabilized the MOF in the presence of an ionic solution. However, the shift in zeta potential to an overall negative surface charge of approximately -50 mV (**Fig. S4c**), and a shoulder in the absorbance spectra of both DNA-coated and DNA_Phos_-coated PCN-222 (**Fig. S4d**) suggest that the salt ageing procedure did not avoid the non-phosphate-modified DNA from binding to the MOF. This proves that the DNA still binds to the MOF through electrostatic interactions between the negatively charged phosphate backbone and the positively charged Zr clusters, even in the presence of an ionic solution.

**Figure S4: Addition of NaCl solution does not prevent the nonspecific interactions between the negatively charged phosphate backbone of DNA and the open metal sites of the MOF.** The incubation between the DNA and DNA_Phos_ with PCN-222 was done in the presence of two different NaCl concentrations, 10 and 250 mM. **a**. Mean hydrodynamic diameter and PDI for PCN-222, DNA@PCN-222 and DNA_Phos_@PCN-222 for both NaCl concentrations. **b**. The corresponding particle size distribution obtained by DLS. **c**. Zeta potential measurements of the samples incubated with 250 mM NaCl. **d**. Absorbance spectra of the materials post incubation with DNA and DNA_Phos_ in the presence of 250 mM NaCl.

### **S5.2 pH titration**

To reduce the charge interactions between the surface of PCN-222 and the phosphate backbone of the DNA, a pH titration was carried out to determine the pH at which the overall surface charge would be neutral or negative, thus minimizing the electrostatic forces. The pH of the PCN-222 solutions in water were adjusted by slowly adding a solution of either 0.1 M HCl or NaOH to obtain pH values that ranged from 3 to 10. Figure S5 shows the effect pH had in the color, size, and zeta potential values of PCN-‑222. When the pH was decreased to very acidic environments (pH = 3 or 4), the color of the solution drastically shifted from the classic pink to green. This change in the color is due to the change in the protonation state of the linker, H_2_TCPP. In neutral or slightly basic conditions, the porphyrin ring remains in its neutral form, and the free carboxylic acid groups are mostly deprotonated. When the environment changes to more acidic conditions, the carboxylic groups are protonated, and the nitrogen atoms in the porphyrinic core gain protons, leading to the formation of a dicationic species. This shift in protonation alters the electronic structure of the porphyrin, which results in a shift of the absorption spectrum towards the green region (**Fig. S6**). Overall, PCN-‑222 seemed to maintain good colloidal stability throughout the whole pH range tested, with the exception of very basic pH values. Between pH values of 3 and 9, the mean hydrodynamic diameter for PCN-222 remained constant at 250 nm, decreasing to 220 nm at pH 10. However, the PDI of PCN-222 at pH 10 increased from 0.1 to 0.3, as observed for the rest of pH range, indicating a less homogeneous system due to aggregation. The zeta-potential remained positive at around +30 mV in acidic conditions and changed to negative values of around -42 mV in neutral to slightly basic conditions. This change in zeta potential is correlated with the protonation/deprotonation behavior of the porphyrinic core. At low pH, the core and any unbound carboxyl group are fully protonated, and the overall positive charge is due to the Zr atoms. As the pH increases, the nitrogen atoms in the porphyrinic core and any free carboxyl groups start to deprotonate, changing the charge of the overall system to negative values.

The effect of pH on the absorbance spectrum of PCN-222 was also studied, as shown in Figure S6. The top part of the figure shows the absorbance spectra of PCN-222 at different pH values immediately after the pH titration experiment, while the bottom part shows the spectra after one hour to determine whether a stabilization period was needed. At acidic pH values (blue), the main absorbance peak is found at 423 nm. As the pH increases, this peak shifts to lower wavelengths, a phenomenon known as blue shift, becoming more intense and sharper, confirming that the electronic environment of PCN-222 is significantly altered by changed in pH.^[58,59]^ At lower wavelengths, the spectra show two broad bands of similar intensity are found at approximately 200 and 240 nm under acidic conditions. As the pH increases, the sharpness and the intensity of the first peak increase, while the second peak diminishes and shifts to lower wavelengths. After one hour, the absorbance spectra show similar trends with slight differences, indicating that the system needs a short stabilization period. The main absorbance peak still shifts to lower wavelengths with increasing pH, but the peak is more pronounced, suggesting that the deprotonated form of PCN-222 becomes fully equilibrated over time. The behavior of the peaks at lower wavelengths remains consistent, with the first peak becoming sharper and more intense, while the intensity of the second peak decreases. In summary, the absorbance spectra of PCN-222 exhibit significant changes with pH, reflecting the protonation and deprotonation processes and confirming that changes in pH result in alterations in the electronic structure, resulting in different color, or surface charge. However, when the DNA and DNA_Phos_ conjugation experiments were carried out at neutral pH values where the zeta potential values were negative, but the MOF retained its colloidal stability, no specific attachment of DNA_Phos_ was observed, and the conjugation of the FAM dye to DNA_Phos_-coated PCN-222 was not successful.

**Figure S5: Effect of pH in the aggregation behavior and zeta potential of PCN-222**. The effect of pH was studied over eight 100 µg/ml solutions of PCN-222. **a**. images of the PCN-222 solutions at different pH values. **b**. Z-average value of PCN-222 at different pH values, and **c**. their corresponding PDI values, obtained with DLS. **d**. Zeta potential measurements for the same pH range. pH was adjusted by gradually adding a diluted HCl or NaOH solution. pH was measured with pH paper, so pH values might not be exact.


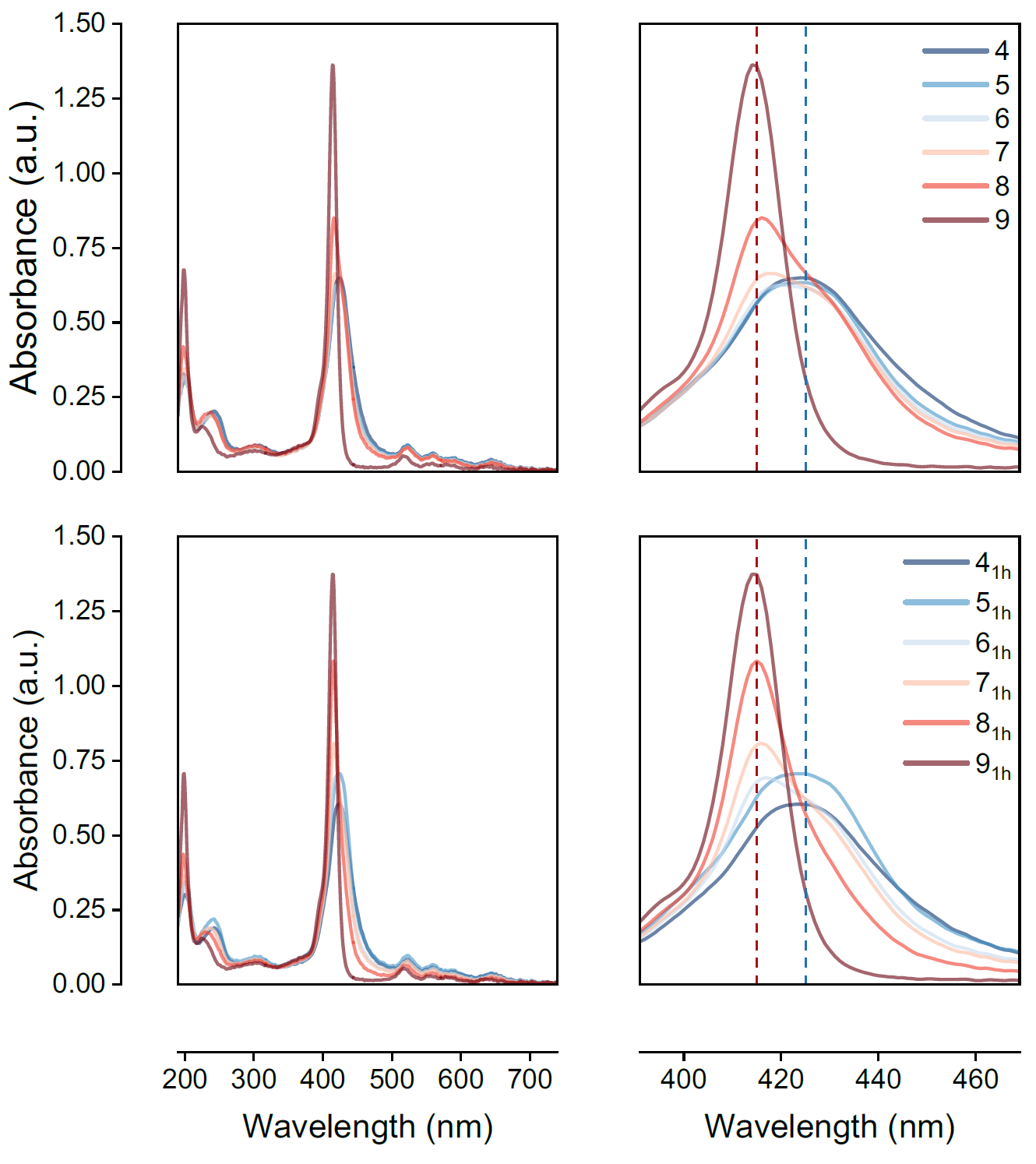


**Figure S6: Effect of pH in the absorbance spectra of PCN-222.** The effect of pH in the absorbance spectra of PCN-222 was studied for different times: immediately after reaction (top) and 1 h after reaction (bottom). The dashed lines correspond to the shift in the main peak from basic pH (red) to acidic pH (blue).

As the salt-ageing procedure and pH titration experiments were not successful in selectively attaching DNA_Phos_ and its conjugation to the FAM dye, the performance of peptide nucleic acid (PNA) was assessed and compare to that of DNA and DNA_Phos_. PNAs are synthetic polymers similar to DNA or RNA but instead of having a phosphate backbone characteristic of DNA, their backbone is made of repeating N-(2-aminoethyl)-glycine units.^[37]^ Despite their different backbone, PNAs are still able to hybridize with DNA, making them an interesting option for avoiding charge interactions.^[40]^ The PNA sequence was designed to be the same as the DNA and DNA_Phos_ and was modified by the addition of a phosphotyrosine base at the end, resulting in phosphate-modified PNA (PNA_Phos_).

## **S6 Characterization of NA-coated PCN-222**


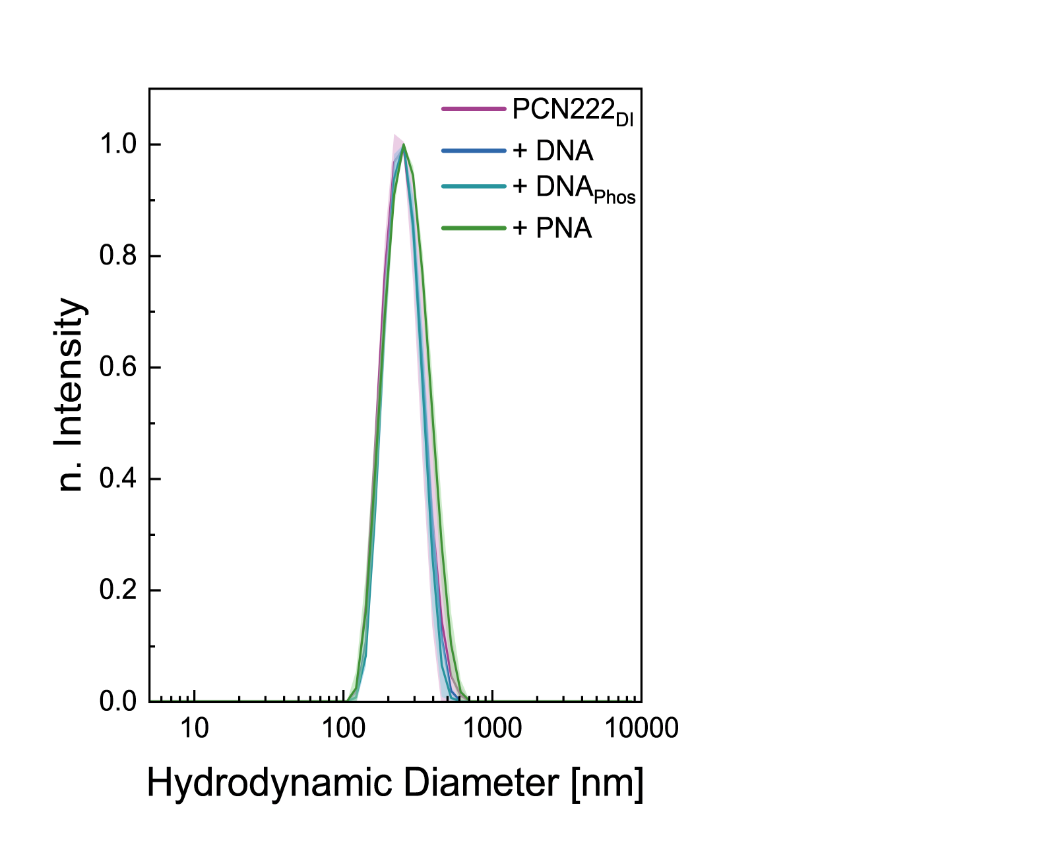


**Figure S7: DLS spectra of NA-coated PCN-222.**


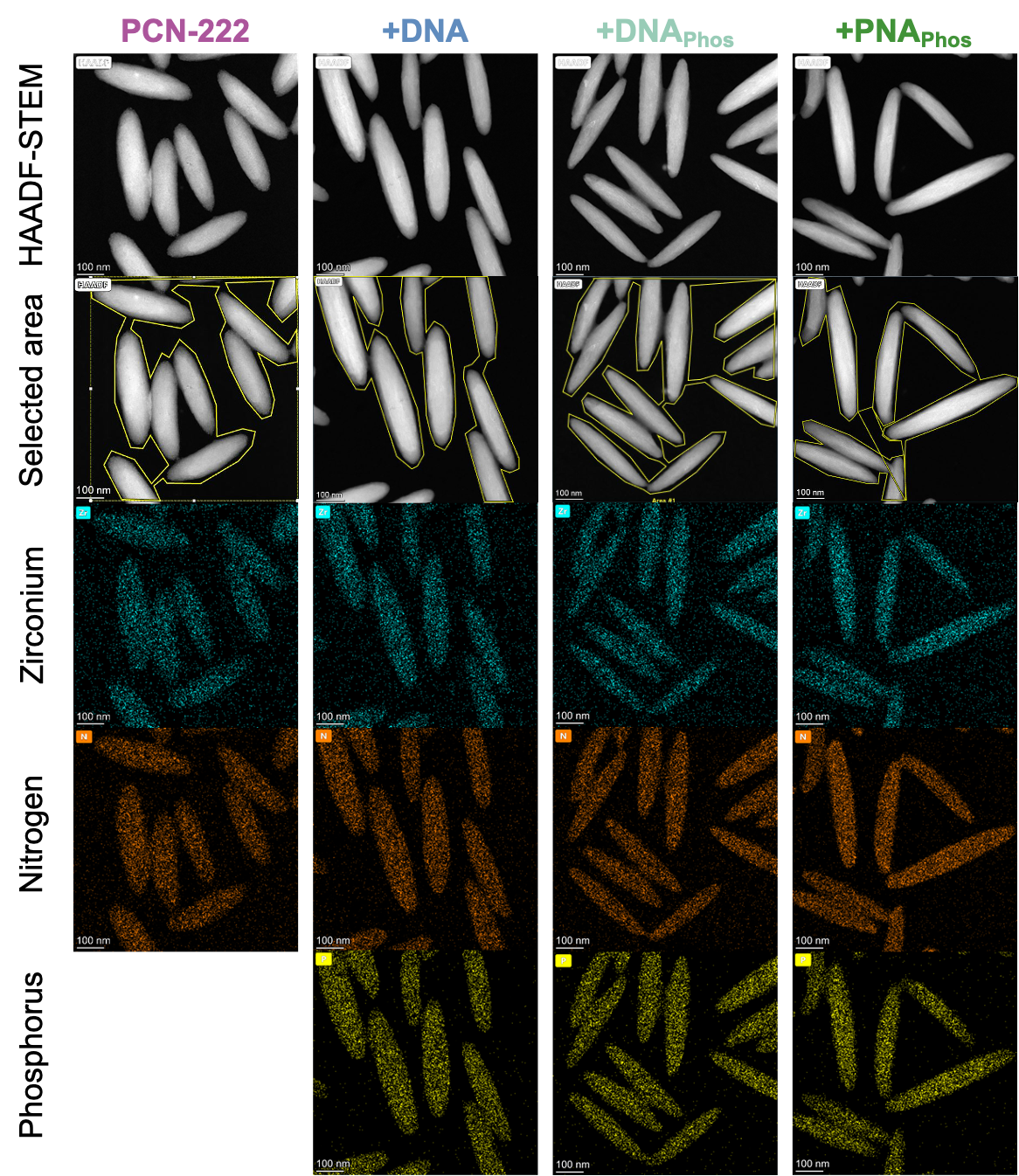


**Figure S8: Energy dispersive X-ray spectroscopy (EDS) analysis for bare, DNA-coated, DNAPhos-coated, and PNAPhos-coated PCN-222.** HAADF-STEM images of PCN-222 and nucleic acid-coated PCN-222, showcasing the selected area that was used to perform EDS area mapping, together with the corresponding elemental maps of zirconium (blue), nitrogen (orange), and phosphorus (yellow). No elemental map of phosphorus is shown for PCN-222 due to the lack of phosphorus in the MOF structure.

**Table S2: Relative atomic fraction values obtained for carbon (C), nitrogen (N), oxygen (O) and zirconium (Zr) using EDS area mapping for bare PCN-222, and DNA-, DNAPhos-, and PNAPhos -coated PCN-222.**

|  | **Zr** | **N** | **P** |
| --- | --- | --- | --- |
| **PCN-222** | 1.31 | 2.66 | 0 |
| **+DNA** | 2.18 | 4.65 | 0.29 |
| **+DNA_Phos_** | 3.11 | 5.06 | 0.24 |
| **+PNA_Phos_** | 2.39 | 8.89 | 0.02 |


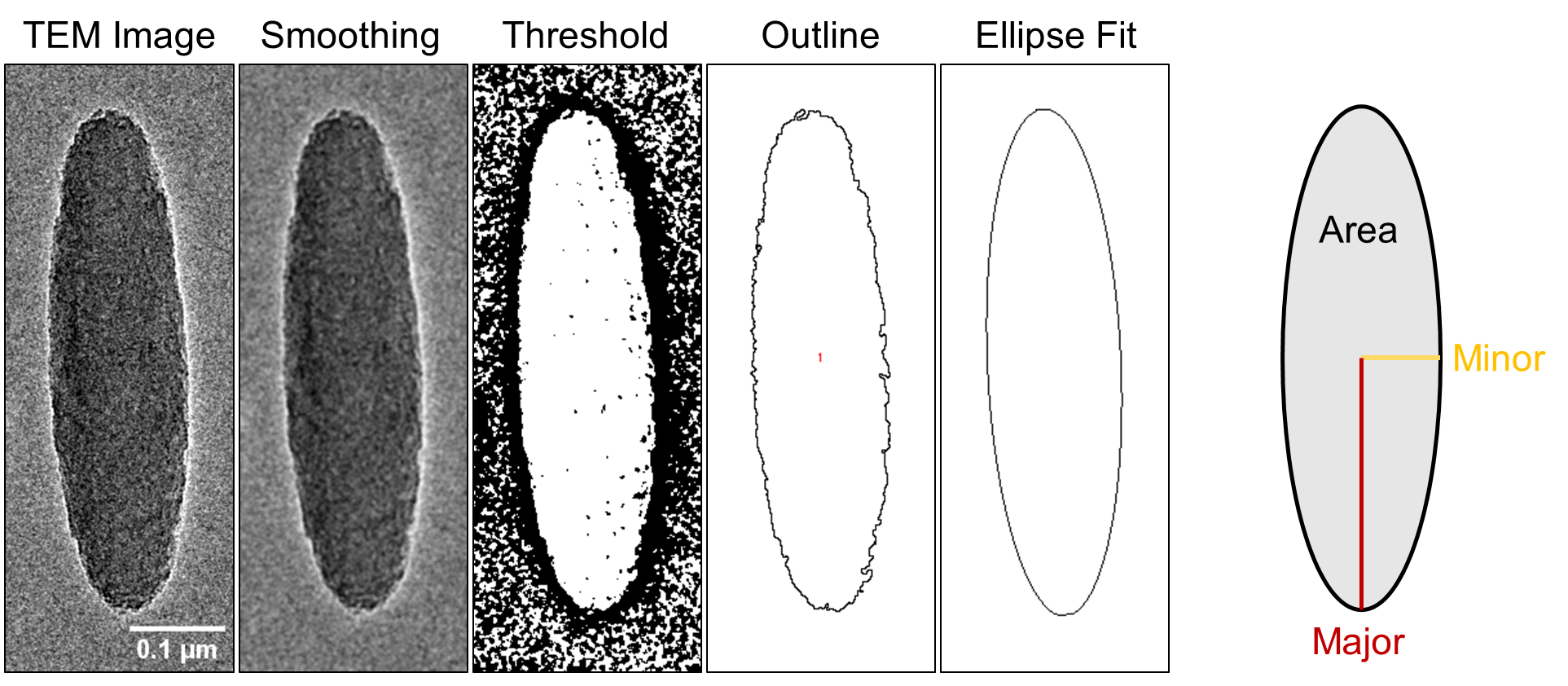


**Figure S9: Fitting strategy of PCN-222 nanoparticles after DNA coating.**


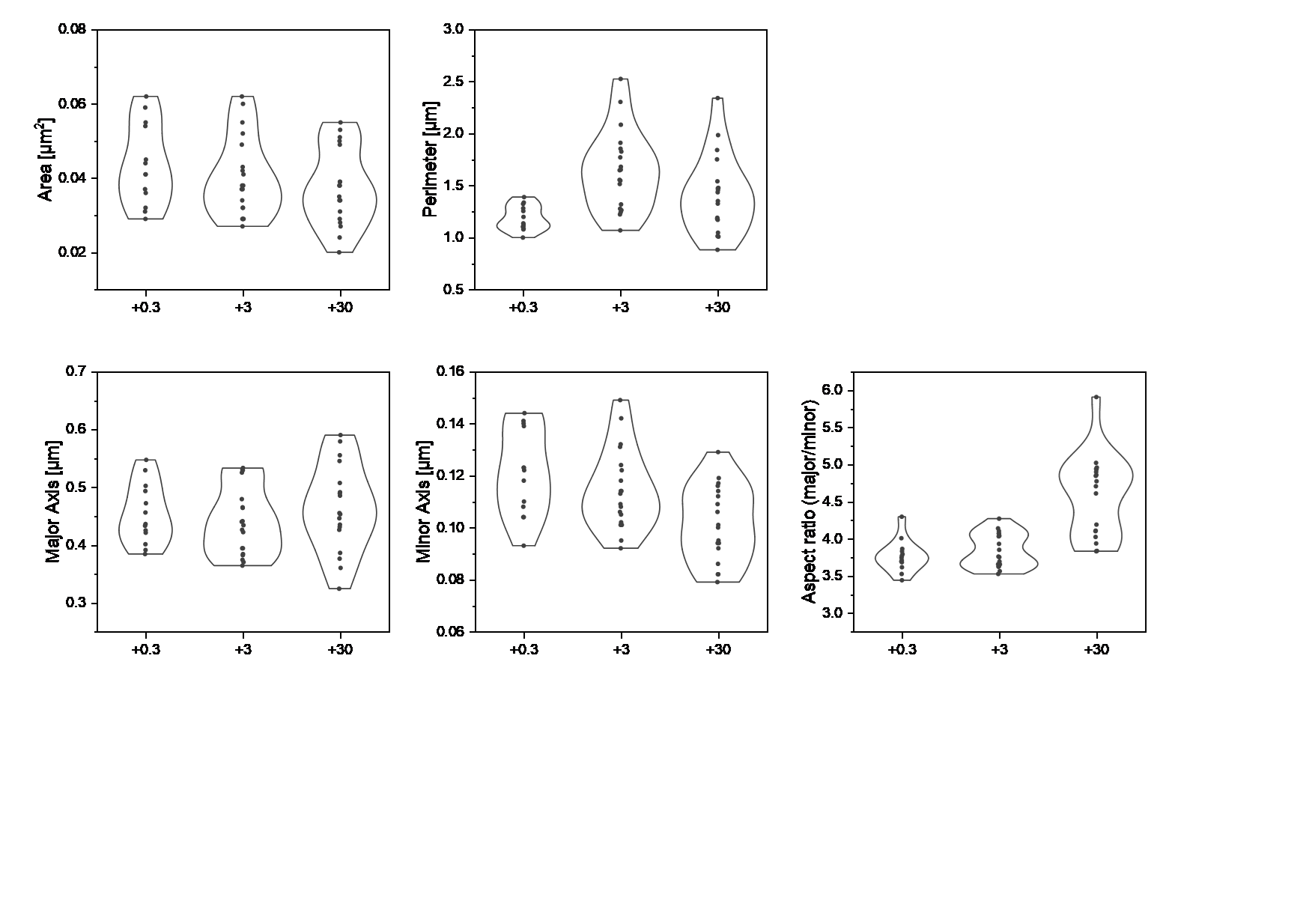


**Figure S10: Effect of increasing DNA_Phos_ concentration on elongation of PCN-222 nanoparticles after fitting.** Extracted from TEM images after incubation of PCN-222 with 0.3, 3, and 30 ng/μL DNA_Phos._


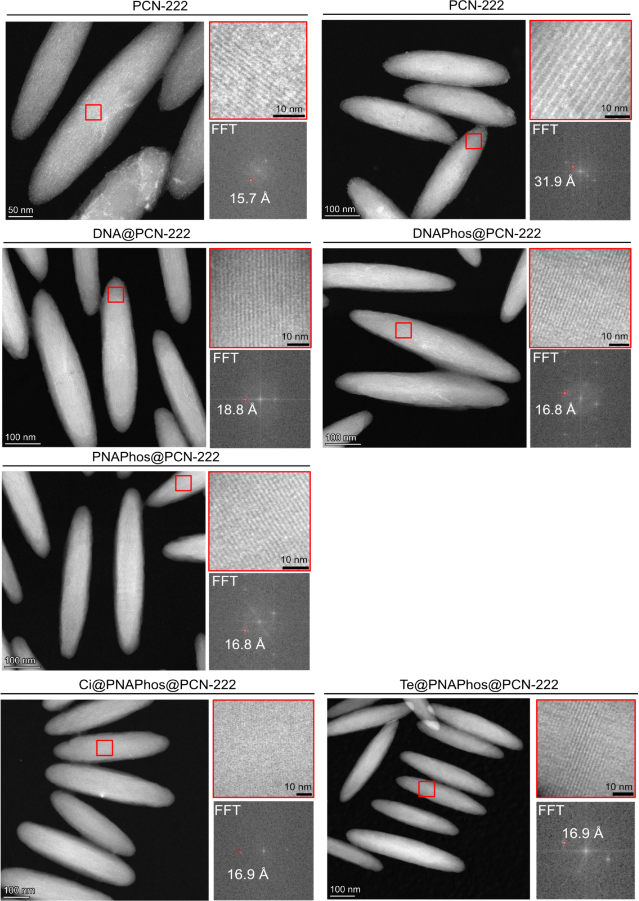


**Figure S11: HAADF-STEM images and corresponding FFT analysis of bare and surface-modified and antibiotic loaded PCN-222 nanoparticles.** Representative low-magnification HAADF-STEM images of two PCN-222 samples with different crystal orientation, DNA@PCN-222, DNAPhos@PCN-222, PNAPhos@PCN-222, Ci@PNAPhos@PCN-222, and Te@PNAPhos@PCN-222 are shown. The red squares indicate the selected regions used for high-magnification lattice fringe analysis. Fast Fourier transform (FFT) patterns obtained from these regions are displayed below each enlargement. The corresponding d-spacings, extracted from the FFT reflections, are indicated in each panel.


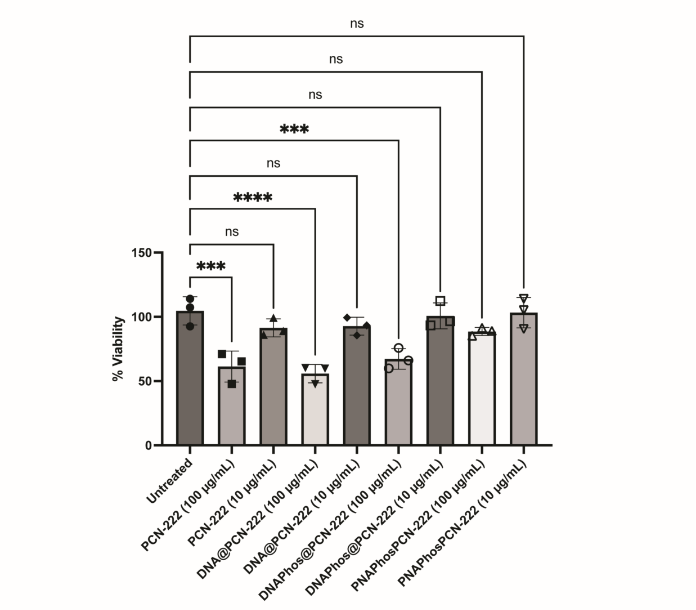


**Figure S12: Viability tests of Wi-38 cells in response to PCN-222 modified with PNAPhos show reduced toxicity compared to bare MOF.** PCN-222 modified with PNAPhos did not show reduced cell viability in Wi-38 cells both at 100 μg/mL and 10 μg/mL after 72 hours of incubation. (n=3 biological repeats). Data represent mean ± SD; ordinary one-way ANOVA with Bonferroni’s multiple comparison test (*p < 0.05, **p < 0.01, ***p < 0.001).

## **S7 Characterization of WGA, Atto-bridge, and WGA-bridge**

**Targeting capabilities of wheat germ agglutinin (WGA)**

The targeting moiety chosen for this study is the model protein Wheat Germ Agglutinin (WGA), a naturally occurring sugar-binding protein (lectin) that plants produce as part of their immune defense against parasites such as bacteria.^[60]^ WGA is a 36 kDa dimer with four binding sites for N-acetyl-D-glucosamine (GlcNAc), a sugar present in the LPS of WT MG1655 cells used in this study, but absent in the LPS of BL21 cells (**Fig. S13**). Thus, MG1655 cells serve as positive controls, while BL21 cells act as negative controls. To verify the targeting capabilities of WGA, an FITC-labeled version was tested against GlcNAc-containing cells (MG1655) and GlcNAc-lacking cells (BL21).


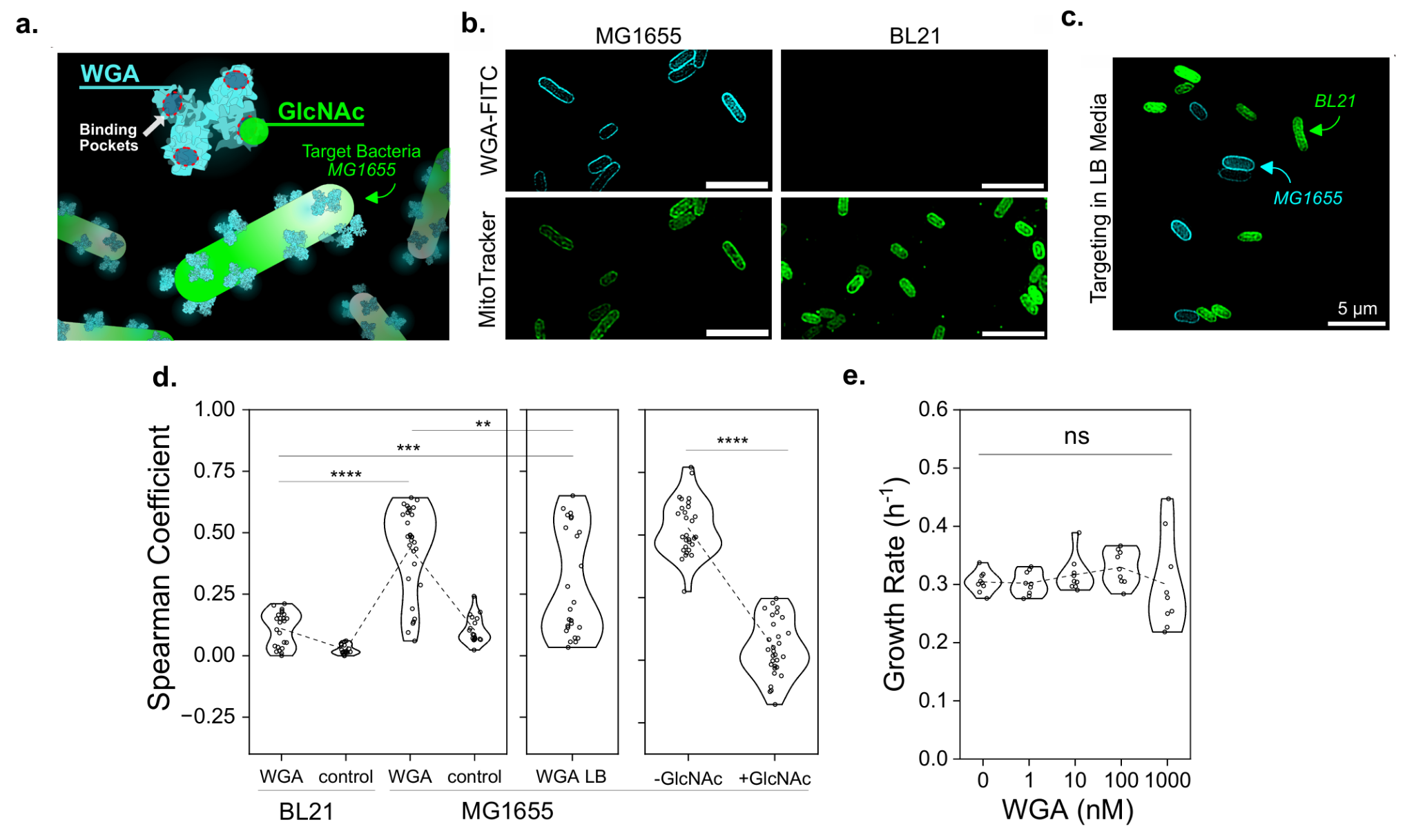


**Figure S13: Targeting capabilities of wheat germ agglutinin (WGA) towards MG1655 bacteria.** a. Schematic of FITC-labelled WGA and its binding to MG1655 bacteria. b. SIM images of FITC-labelled WGA (cyan) and bacteria (green). c. SIM image proving specific targeting of MG1655 by WGA in a co-culture of MG1655 and BL21. d. Co-localization analysis for BL21 and MG1655 bacteria. e. Growth rate of MG1655 in the presence of difference concentrations of WGA.

As shown in the representative SIM images in Figure S11b,c, MG1655 cells were labeled by WGA, while BL21 cells remained unlabeled. Co-localization analysis between Mitotracker Deep Red-labeled cell membranes and FITC-labeled WGA showed significant overlap for MG1655, confirming the successful binding of WGA to MG1655 cells. In contrast, BL21 cells showed insignificant overlap, confirming the absence of WGA binding due to the lack of GlcNAc in its LPS layer. In a co-culture of MG1655 and BL21, FITC-labeled WGA continued to selectively target MG1655, demonstrating its potential as a precise targeting moiety even in more complex environments, such as LB media (**Fig. S13c,d**). The binding specificity to GlcNAc was further confirmed through a glycan inhibition study, in which WGA was co-incubated with a saturating amount of GlcNAc and bacteria, resulting in significantly lower co‑localization between cells and WGA (**Fig. S13d**). Lastly, WGA concentrations up to 1 µM did not significantly affect bacterial growth, confirming its suitability as a targeting moiety that does not interfere with bacterial viability (**Fig. S13e**).

**Targeting Bridge Design and Assembly**

Here, a DNA bridge was explored as a highly customizable attachment strategy of WGA to PCN-222. We chose a DNA-based assembly, as it allows for easy strand exchange and the addition of commercial functionalizations that enable visualization and the attachment of proteins (**Fig. S14a**).

The targeting bridge design here relies on a three-part system (**Fig. S14a**). First, a ssDNA designed to bind to PCN-222. Second, a bridge DNA sequence of approximately 20.4 nm (60 Nucleotides (N)) that binds to the cholesterol modified ssDNA. The bridge sequence is constructed from two 40 N ssDNA sequences that hybridize in the middle through 20 N complementarity resulting in two overhangs on both sides of 20 N length. This length was chosen to construct stable DNA that provides sufficient spacing to prevent steric hindrance of the WGA when attached to the PCN-222 surface. Lastly, the WGA attached to a ssDNA with the routinely performed click reaction between DBCO and azide groups. Azide-modified DNA is commercially available, while proteins can be purchased or post-translationally modified to carry DBCO.

**Gel electrophoresis**

To determine whether the formation of the Atto561-bridge was successful, an agarose gel was performed (**Fig. S14b**). The second lane of the gel shows a band that corresponds to the DNA strand that is carrying the Atto561 dye (B_Atto_). In the third lane, a band is detected at a higher molecular weight than that of B_Atto_, confirming that successful hybridization of the complementary B_1_ and B_2_ DNA strands took place. Finally, the band on the fourth lane corresponds to the Atto-bridge (B_A_), and since the band is located at a higher molecular weight than B_1_B_2_, the complete assembly of the Atto-bridge from the hybridization of B_1_B_2_ and B_Atto_ is confirmed.

**SDS-Page**

The success of the conjugation between a ssDNA sequence and WGA via click‑chemistry was confirmed by SDS-PAGE (**Fig. S14c**), as an increase in approximately 9 and 18 kDa (columns 2 and 3) was observed when compared to the WGA (column 1). This suggests that either one or two ssDNA were attached, respectively.


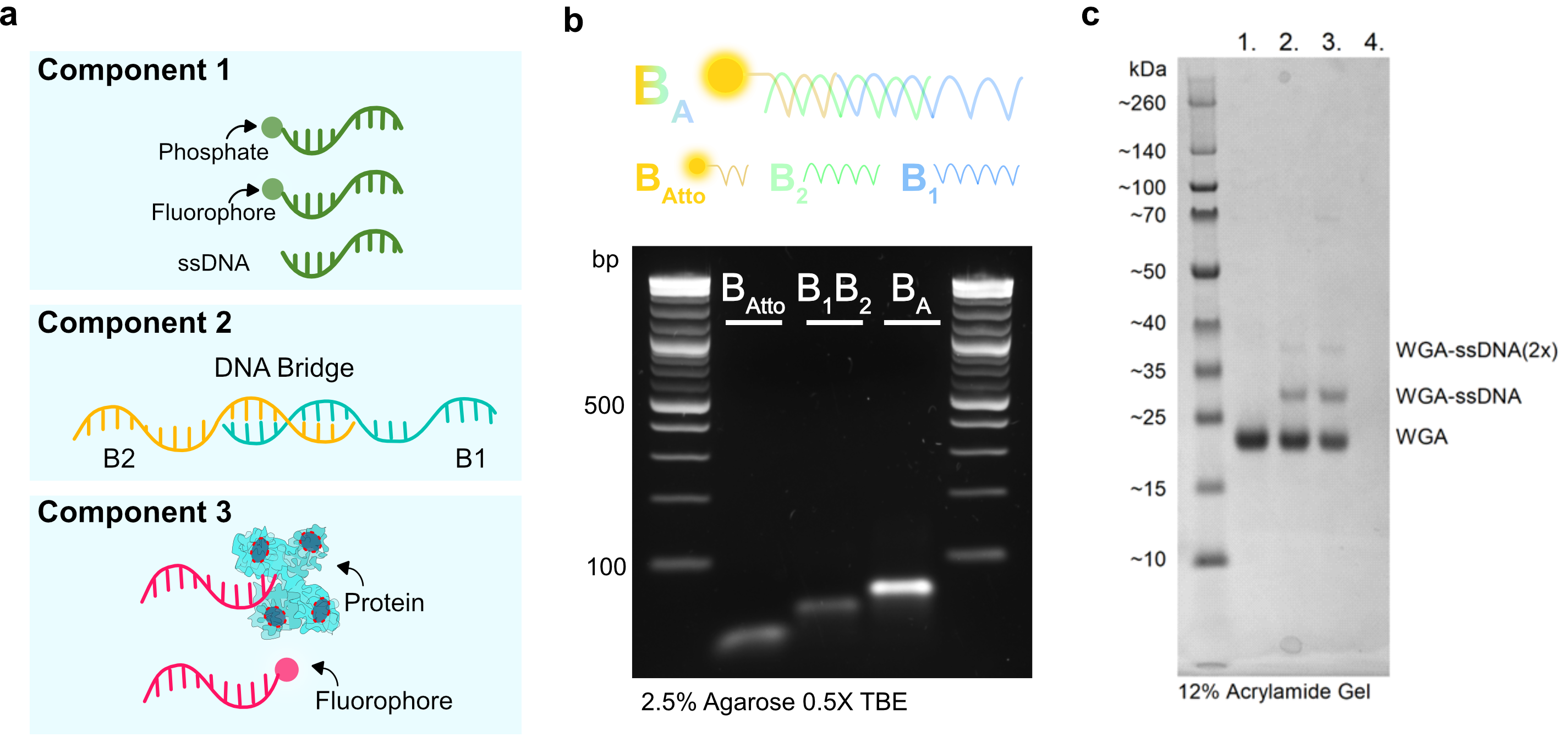


**Figure S14: Gel electrophoresis of Atto561 bridge and WGA-ssDNA.** **a**. Illustration of a three-component DNA system, referred to as the targeting bridge, which consists of an NP linker sequence or fluorescent label, a 60 nucleotide DNA bridge that connects the first component with the WGA of fluorophore linked to ssDNA. **b**. Agarose gel of the individual bridge components (B_Atto_ and B_1_B_2_) and their assembly (B_A_). **c**. Acrylamide gel of WGA-ssDNA. Numbers correspond to (1) WGA, (2) WGA:DNA (1:3 molar ratio) 12 h RT, (3) WGA:DNA (1:9 molar ratio) 12 h 37 ºC, (4) DNA.

**Targeting of WGA after hybridization with Atto561-bridge**


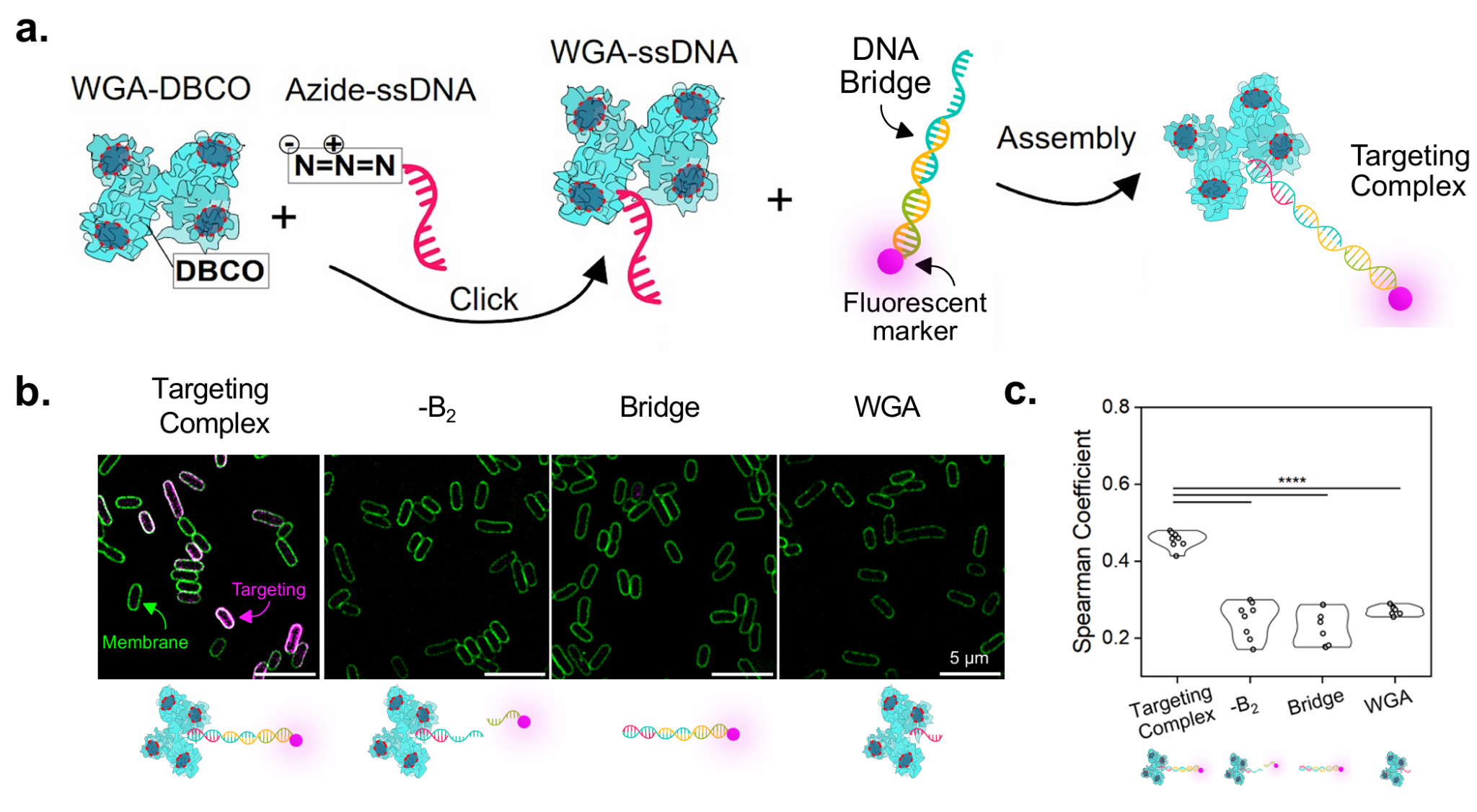


**Figure S15: Assembly of the targeting complex with WGA.** a. Schematic of the assembly of the targeting moiety. b. SIM images of MG1655 (green) and Atto561conjugated WGA (pink) in the presence of the targeting complex, targeting complex without B2, Atto-bridge, and WGA. c. co-localization analysis of the targeting unit (assembly) and its negative controls (-B2, bridge, and WGA).

## **S8 Assessment of drug-loaded PCN-222**


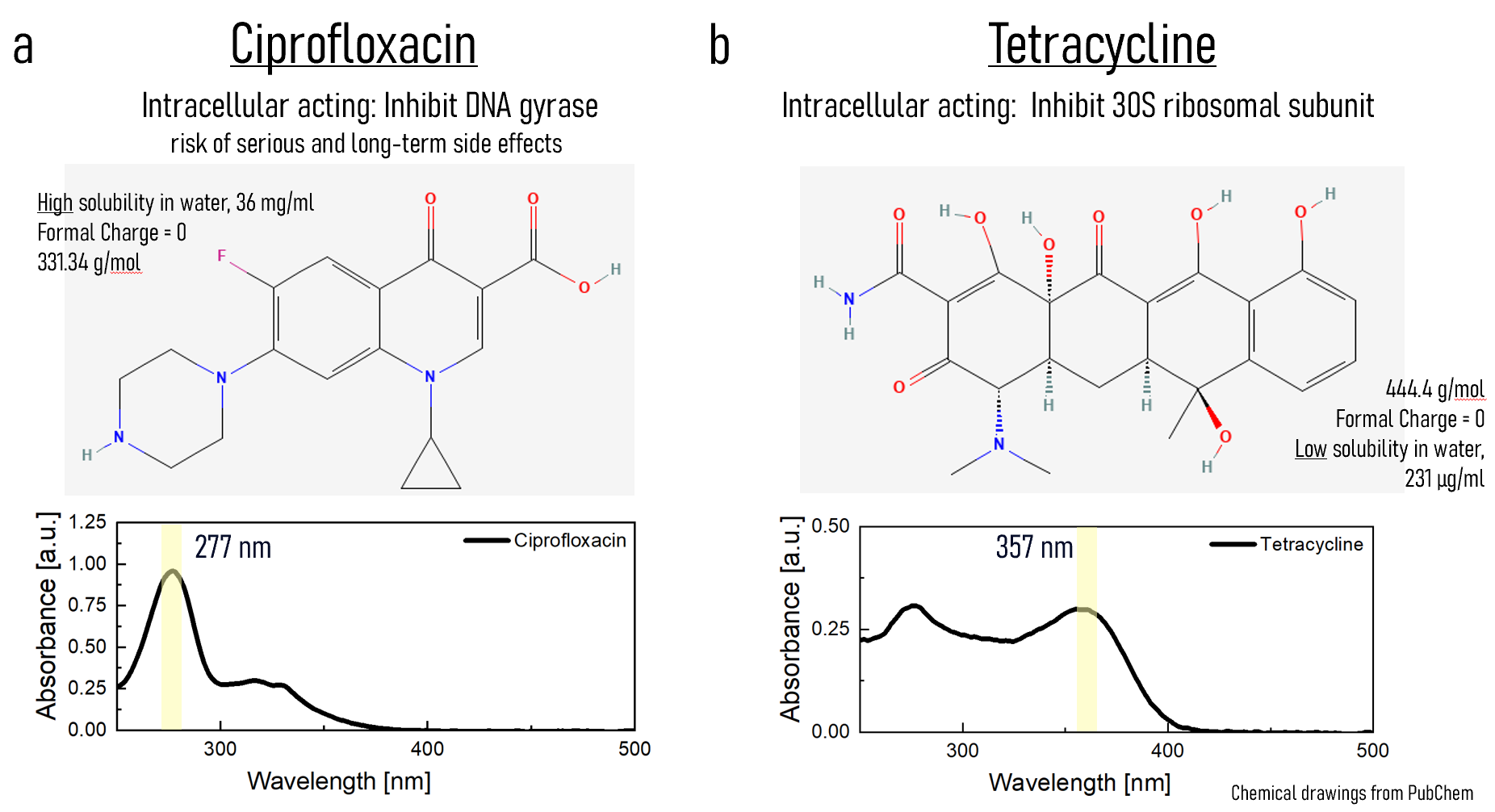


**Figure S16: Properties of different antimicrobials.** Chemical structure, molecular weight, charge, and absorption spectra for a. ciprofloxacin and b. tetracycline. Chemical drawings were extracted from PubChem.


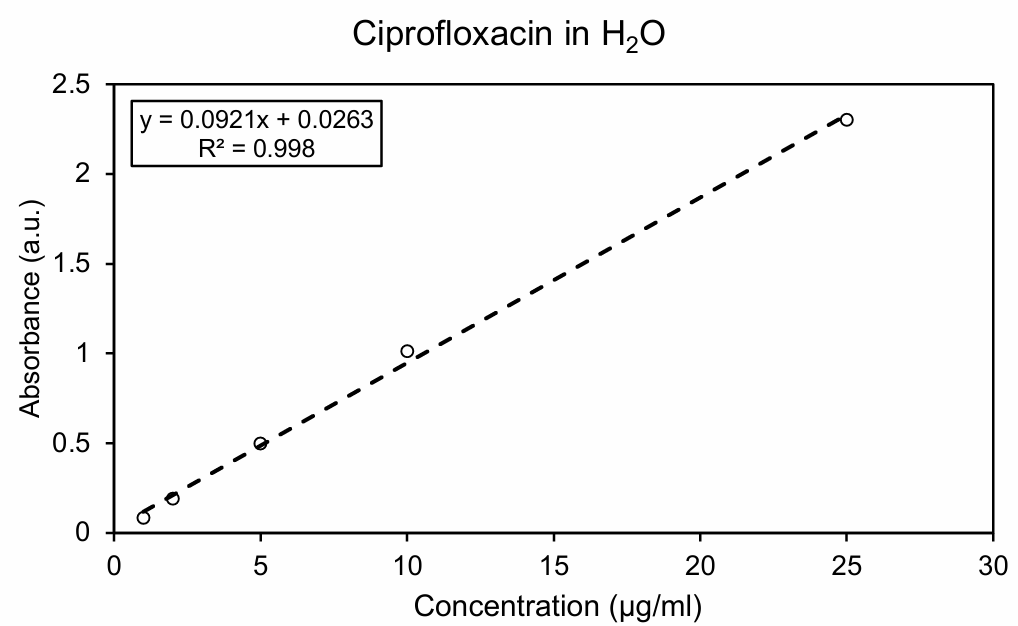


**a.**

**b.**


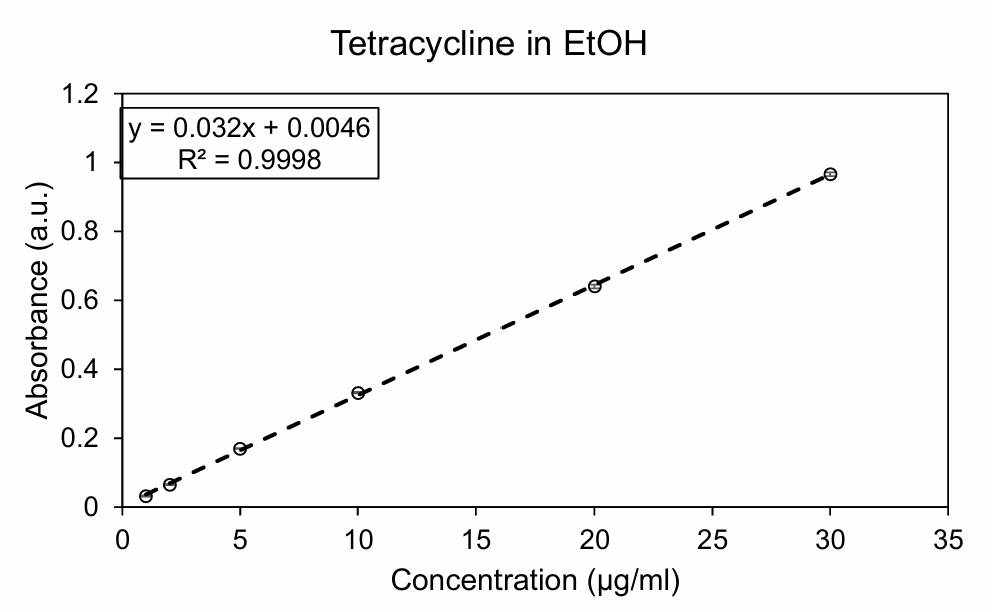


**c.**


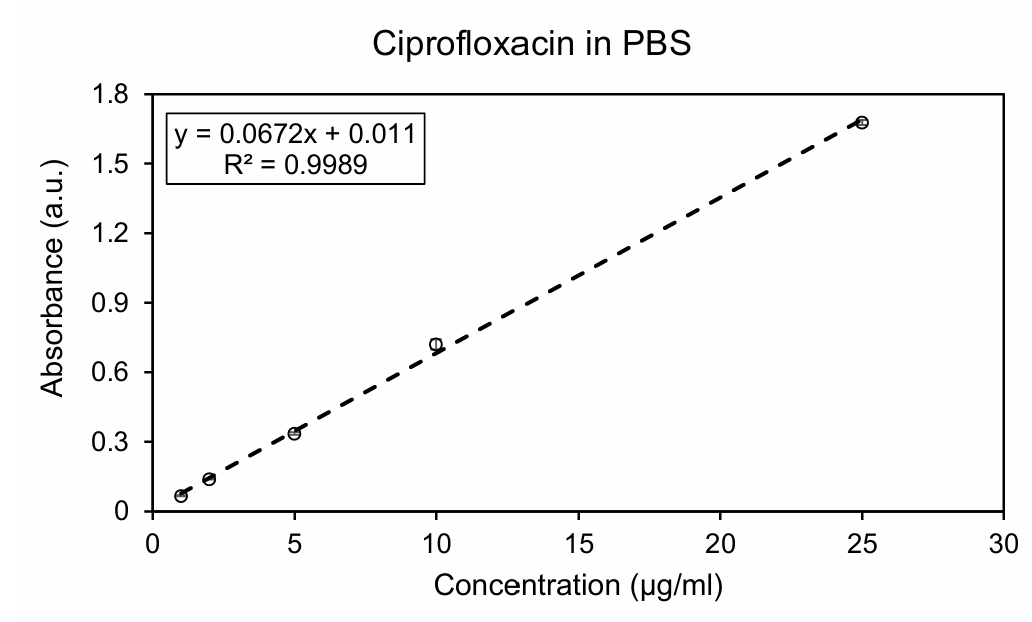


**Figure S17: Calibration curves of antimicrobials, obtained by UV-Vis**. **a**. Ciprofloxacin in H_2_O. **b**. Tetracycline in EtOH. **c**. Ciprofloxacin in PBS.


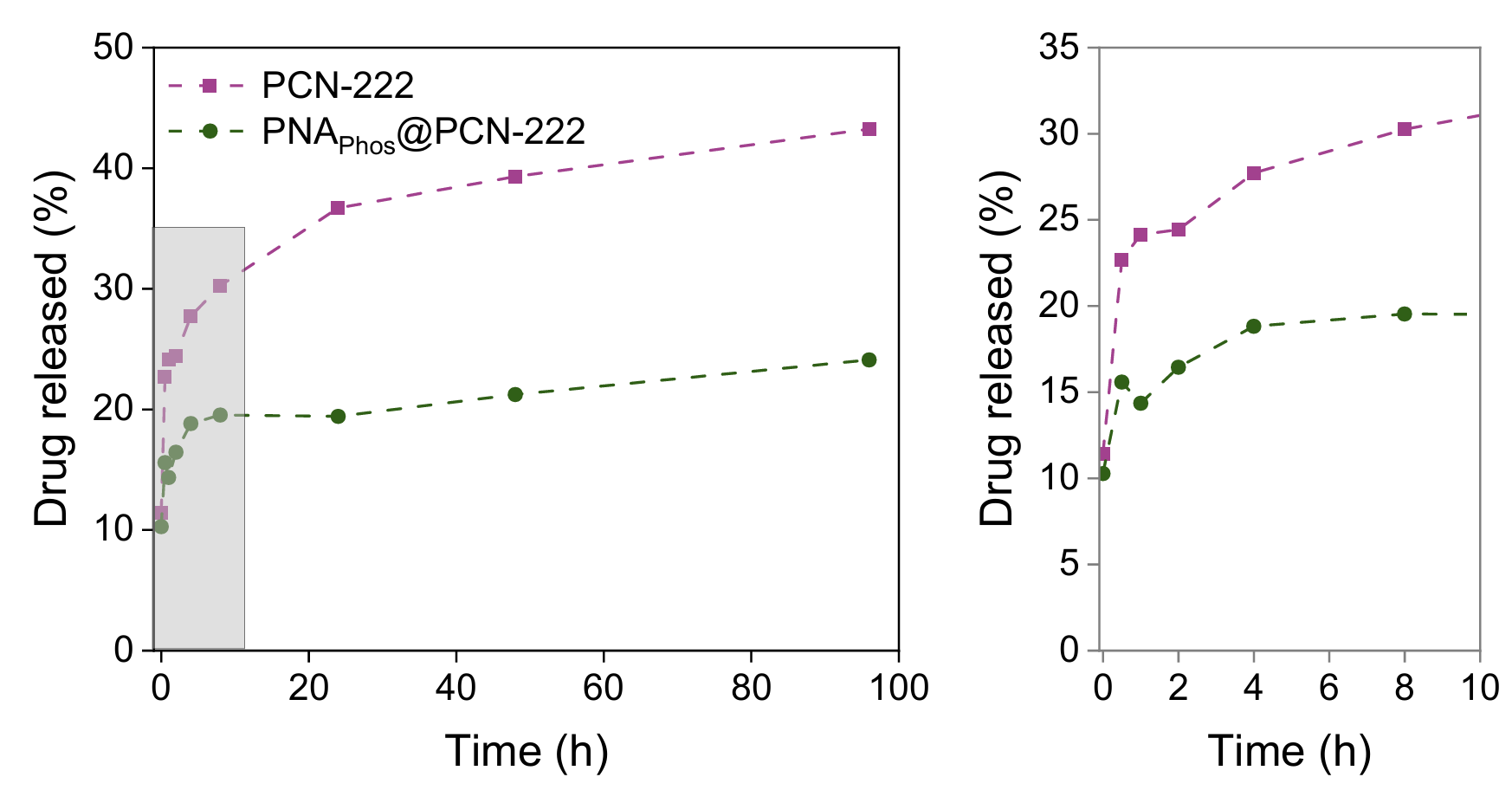


**Figure S18: Drug-release studies of ciprofloxacin.** The release of ciprofloxacin from PCN-222 (pink) and PNA_Phos_-coated PCN-222 (green) was carried out in PBS for 96 hours.

After the successful encapsulation of the drugs was confirmed, Te@PCN-222 was solvent exchanged with water twice via centrifugation and resuspension, before coating both Ci@PCN-222 and Te@PCN-222 with DNA. After the coating process was carried out, the effect of the free antimicrobial drugs, DNA@PCN-222, and the DNA-coated and drug-loaded DNA@Ci@PCN-222 and DNA@Te@PCN-222 on the growth curves of bacteria was studied. These experiments were performed without the targeting bridge to study the effectiveness of antibiotics encapsulated in DNA-coated MOFs. Figure S17 shows the growth curves and growth rates of the drug-loaded and coated materials, along with the free drugs and the DNA-coated PCN-222 at different concentrations. In the presence of DNA@PCN-222 (**Fig. S19**), the growth of MG1655 bacteria was not affected at concentrations of DNA@PCN-222 as high as 10 μg/ml, confirming the non-toxic nature of the nanocarrier. On the other hand, both drugs exhibited bactericidal or bacteriostatic properties towards MG1655 bacteria (**Fig. S19b,d**). Ciprofloxacin killed all the planktonic bacteria at concentrations of 0.1 and 1 μg/ml, but bacterial growth was unaffected at concentrations of 0.01 and 0.001 μg/ml. Tetracycline, however, was less effective and only inhibited bacterial growth at the highest concentration tested (10 μg/ml). As the drug concentration decreased, bacterial growth rates increased. When the drugs were encapsulated in PCN-222, their performance varied when compared to the free drug. DNA@Ci@PCN-‑222 showed a similar trend to free ciprofloxacin, killing all bacteria at high concentrations (1 μg/ml) but having no significant effect at low concentrations (0.01 and 0.001 μg/ml). The main difference appeared at the middle concentration of 0.1 μg/ml, where free ciprofloxacin killed all the bacteria while its encapsulation in the MOF partially slowed down the initial bacterial growth. This suggests that at high concentrations, the slow release of the drugs from the pores of the MOF is sufficient to kill the bacteria, whereas at the middle concentration it is not.


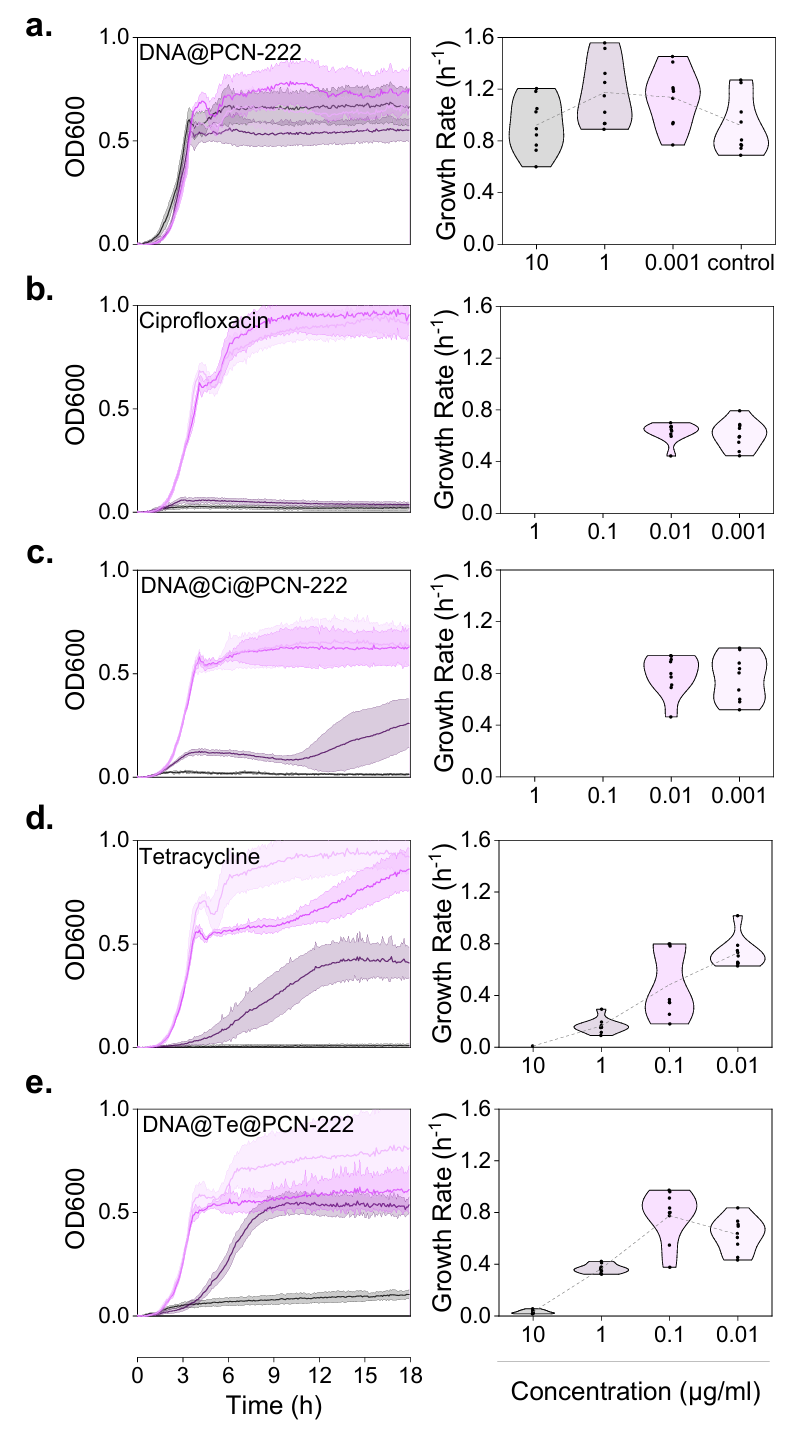


**Figure S19: Toxicity of PCN-222, ciprofloxacin, tetracycline, and drug-loaded PCN-222.** Optical density plots displaying the growth of bacteria over time and the corresponding growth rate for different concentrations of **a**. PCN-222, **b**. Ciprofloxacin, **c**. Ciprofloxacin-loaded PCN-222, **d**. Tetracycline, and **e**. Tetracycline-loaded PCN-222. Drug-loaded molecules were coated with DNA.


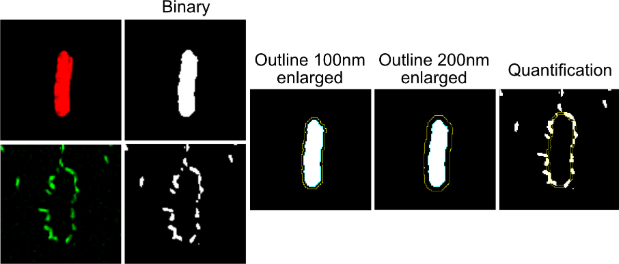


**Figure S20: Image Processing pipeline for the quantification of PCN-222 bacteria surface coverage.** The targeting efficiency between the different PCN-222 coatings was determined as the percentage of the bacterial outline associated with PCN-222. An ImageJ-based image processing pipeline was developed to determine the coverage percentage. The images were first split into cytoplasmic staining and PCN-222 signal channels, then thresholded and converted to binary images with white (255) signals against a black (0) background. The cytoplasmic outline was selected and enlarged by 100 nm to approximate the outer bacterial cell envelope border and saved as a region of interest (ROI). This outline was further enlarged by another 100 nm and saved as a second ROI. The area between these two ROIs was used to measure surface coverage. The ROIs were applied to the thresholded PCN-222 image, the percentage of white (255) pixels within this region was quantified (ranging from 0 to 1, with 1 indicating full surface coverage) and plotted as “surface coverage”.


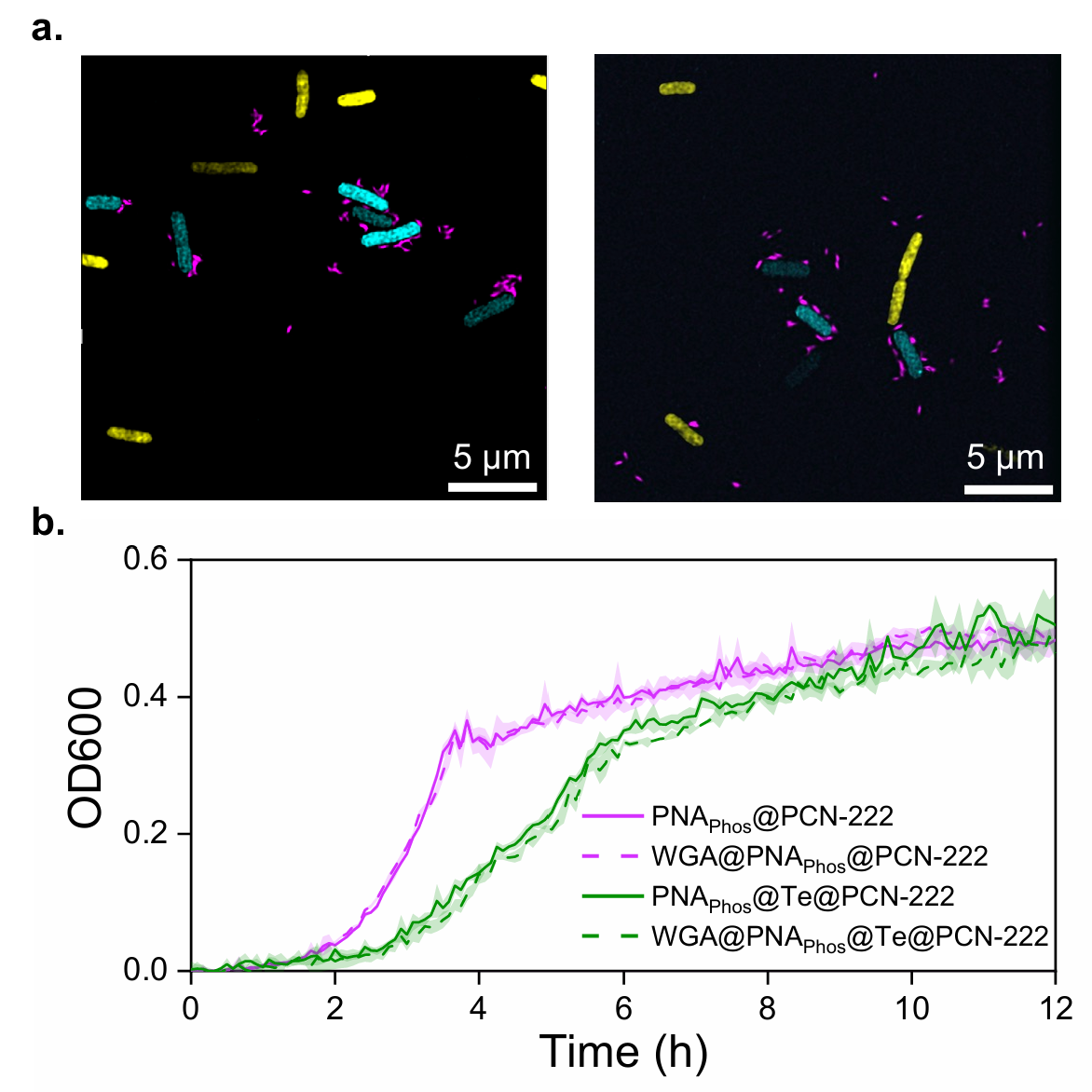


**Figure S21: Bacteria targeting of PNA_Phos_-coated and tetracycline-loaded PCN-222 after hybridization with WGA bridge.** Growth curves of bacteria in the presence of unloaded PCN-222 (pink) and tetracycline-loaded PCN-222. Dashed lines represent the MOF with the targeting complex.
